# Image-based model of the spectrin cytoskeleton for red blood cell simulation

**DOI:** 10.1371/journal.pcbi.1005790

**Published:** 2017-10-09

**Authors:** Thomas G. Fai, Alejandra Leo-Macias, David L. Stokes, Charles S. Peskin

**Affiliations:** 1 John A. Paulson School of Engineering and Applied Sciences, Harvard University, Cambridge, Massachusetts, United States of America; 2 Leon H. Charney Division of Cardiology, New York University School of Medicine, New York, New York, United States of America; 3 Skirball Institute of Biomolecular Medicine, Department of Cell Biology, New York University School of Medicine, New York, New York, United States of America; 4 Courant Institute of Mathematical Sciences, New York University, New York, New York, United States of America; University of Pennsylvania, UNITED STATES

## Abstract

We simulate deformable red blood cells in the microcirculation using the immersed boundary method with a cytoskeletal model that incorporates structural details revealed by tomographic images. The elasticity of red blood cells is known to be supplied by both their lipid bilayer membranes, which resist bending and local changes in area, and their cytoskeletons, which resist in-plane shear. The cytoskeleton consists of spectrin tetramers that are tethered to the lipid bilayer by ankyrin and by actin-based junctional complexes. We model the cytoskeleton as a random geometric graph, with nodes corresponding to junctional complexes and with edges corresponding to spectrin tetramers such that the edge lengths are given by the end-to-end distances between nodes. The statistical properties of this graph are based on distributions gathered from three-dimensional tomographic images of the cytoskeleton by a segmentation algorithm. We show that the elastic response of our model cytoskeleton, in which the spectrin polymers are treated as entropic springs, is in good agreement with the experimentally measured shear modulus. By simulating red blood cells in flow with the immersed boundary method, we compare this discrete cytoskeletal model to an existing continuum model and predict the extent to which dynamic spectrin network connectivity can protect against failure in the case of a red cell subjected to an applied strain. The methods presented here could form the basis of disease- and patient-specific computational studies of hereditary diseases affecting the red cell cytoskeleton.

## Author summary

Red blood cells are responsible for delivering oxygen to tissues throughout the body. These terminally differentiated cells have developed a fascinating flexibility and resiliency that is critical to navigating the circulatory system. Far from being rigid bodies, red blood cells adopt biconcave disk shapes at equilibrium, parachute-like shapes as they move between large vessels and small capillaries, and more extreme shapes as they traverse the endothelial slits of the spleen. Understanding the remarkable mechanical properties that allow red cells to experience such large deformations while maintaining structural integrity is a fundamental question in physiology that may help advance treatments of genetic disorders such as hereditary spherocytosis and elliptocytosis that affect red cell flexibility and can lead to severe anemia. In this work, we present a model of the red blood cell cytoskeleton based on cryoelectron tomography data. We develop an image processing technique to gather statistics from these data and use these statistics to generate a random entropic network to model the cytoskeleton. We then simulate the behavior of the resulting red blood cells in flow. As we demonstrate through simulations, this method makes it possible to examine the consequences of changes in microstructural properties such as the rate of cytoskeletal remodeling.

## Introduction

Red cells possess a lipid membrane and cytoskeleton that together enclose a viscous cytoplasm characterized by a high concentration of hemoglobin. The elastic properties of the cell can be separated into contributions from the lipid bilayer, which supplies bending rigidity and resistance to local changes in area, and from the cytoskeleton, which is a polymer network of spectrin tetramers connected at actin-based junctional complexes that supplies shear resistance. In previous work [1], we used a continuum neo-Hookean model [2] to describe the coupled membrane-cytoskeleton system, and we simulated the behavior of red cells in flow using the immersed boundary method, a numerical method for fluid-structure interaction problems [3]. However, applying the continuum approach to both the lipid membrane and cytoskeleton can be inadequate for certain applications because of the wide range of scales needed to describe the system (e.g. the phospholipids that make up the membrane are approximately 8 Å apart [4], whereas the average size of spectrin tetramers in the cytoskeleton is about 50 times larger [5]). On the one hand, continuum models correctly predict that red cells “remember” the positions of their biconcave dimples [6], but on the other hand there is evidence that the cytoskeleton is constantly remodeling [7] so that the reference configuration changes over time, a property not taken into account in standard neo-Hookean continuum models. The sensitivity of measuring the shear modulus to the particular experimental setup [8, 9] also suggests that neo-Hookean models of the cytoskeleton may be overly simplistic. Characterizing the cytoskeletal mechanics in detail, including the nature of network remodeling, is crucial for understanding the red cell’s exceptional deformability [7] and for explaining the experimental effects of repeated osmotic swelling and shrinking on red cell elasticity [10].

In light of these issues, the approach taken here is to build a model based on the molecular cytoskeletal structure. In particular, we retain the continuum description of the lipid membrane but replace the continuum cytoskeletal model with a discrete one. Significant steps in this direction have already been made, starting with Boal’s early work involving Monte Carlo simulations of small regions of the cytoskeleton that suggested the importance of volume exclusion effects for polymer models [11]. Later, Discher et al. studied the mechanical response of red cells during micropipette aspiration experiments using a discrete cytoskeleton model [12]. More recently, the group of Suresh simulated whole cells using a detailed cytoskeleton model that considered interactions between spectrin monomers via molecular dynamics and allowed for network reorganization [13–15]. Cytoskeletal structure has also been incorporated into composite models, in which the membrane and cytoskeleton are treated as distinct components, and which explicitly model the vertical connections between membrane and cytoskeleton that are affected in conditions such as hereditary spherocytosis [16–18]. In such composite models, the cytoskeleton is able to undergo changes in density, an effect that has been observed during micropipette aspiration [19]. Further, Pivkin et al. used coarse-graining techniques to calculate the spectrin network’s effective shear modulus as a function of the number of degrees of freedom used to represent the red cell surface [20]. Although some simulations of red cell cytoskeletons assume a hexagonal topology, our work allows for the irregular topology, as do e.g. Saxton [21], Hansen et al. [22], Li et al. [13], and Gov [23].

Here, we propose a mesoscopic model of the cytoskeleton that we use for whole cell immersed boundary method simulations. Although others have used random graph models of the spectrin cytoskeleton, a novel feature of our work (to the best of our knowledge) is that we derive the statistical properties of the random graph from 3D electron tomographic images. Representing the cytoskeleton as an explicit polymer network allows us to investigate the effect of changes at the microscopic level, such as network remodeling, in a relatively straightforward manner. This approach allows us to investigate changes in the horizontal connections within the cytoskeleton, complementing the studies cited above that model the vertical connections between the cytoskeleton and membrane. We use the immersed boundary method to account for the coupling of the red cell to the surrounding fluid. The immersed boundary method has previously been applied to many problems in cellular mechanics, including cytoskeletal mechanics [24, 25], tumor cell adhesion [26], flagellar motion [27, 28], and cytoplasmic streaming [29].

This article is structured as follows: we first describe an algorithm to generate random graphs on surfaces with a specified node density, edge length distribution, and mean number of edges per node. In our model, the edges represent spectrin polymers that span junctional complexes, and we treat these polymers as entropic springs. We next show, using a simple two-dimensional geometry, that properly-initialized random networks exhibit elastic responses that are in good agreement with continuum models. No parameter fitting is needed to obtain this agreement; the entropic spring constant and parameters needed to generate the random graph have been measured in experiments described in the literature. Next, we introduce a segmentation algorithm that we use to extract geometrical information from 3D images generated by electron tomography. We apply this segmentation algorithm to a tomogram and use the resulting distributions as geometrical and statistical constraints for our model. We generate a random cytoskeleton on a triangulated surface representing a whole red cell and perform three-dimensional immersed boundary simulations to study the behavior of red blood cells in a prescribed shear flow. We further show how network remodeling can be included in this model and consider its effects on the frequency of spectrin polymer failure when the cell is subjected to a prescribed strain, as can be done in optical tweezer experiments.

## Methods

### Generating the cytoskeleton

We represent the cytoskeleton as a random graph on a surface, with the junctional complexes serving as nodes and the spectrin tetramers serving as edges. The node positions are drawn from a uniform distribution with respect to area, and nodes are randomly connected by edges using an algorithm that recovers the prescribed end-to-end length distribution. Here, we describe this two-step process in the case of *N* total nodes.

**Step 1**. Choose *N* points independently on a surface with surface area *S* from the uniform distribution with respect to area.
**Step 2**. For each pair of points *i* and *j*, make a connection independent of any other pair with probability *p*(*D*_*ij*_), where *D*_*ij*_ is the distance between nodes *i* and *j* and *p*(*D*) is some given function satisfying 0 ≤ *p*(*D*) ≤ 1.

We assume that there is a maximum edge length *D*_max_ such that *p*(*D*) = 0 for *D* > *D*_max_. As a result of Step 1, there is a probability density function *ϱ*(*D*) such that

$$\begin{array}{c} \int_{a}^{b}\varrho \left(D\right)dD=\text{Pr}\left(D_{ij}\in \left(a,b\right)\right), \end{array}$$ (1)

where the right-hand side denotes the probability that *D*_*ij*_ is in the interval (*a*, *b*). Note that *ϱ*(*D*) gives the distribution of distances between arbitrary nodes on the surface independent of whether those nodes are connected. The probability *P* that there is an edge between any given pair of nodes is given by

$$\begin{array}{c} P=\int_{0}^{D_{\text{max}}}p\left(D\right)\varrho \left(D\right)dD. \end{array}$$ (2)

Since an arbitrary pair of points is assigned an edge with this probability, independent of any other pair of points, the probability that exactly *k* edges touch any given node is given by the binomial distribution

(N-1k)Pk(1-P)N-1-k, (3)

which has mean

$$\begin{array}{c} \mu =(N-1)P. \end{array}$$ (4)

We are interested in *N* large and *P* small, so that (3) is well approximated by the Poisson distribution

$$\begin{array}{c} \frac{\mu^{k}}{k!}e^{-\mu }. \end{array}$$ (5)

Another quantity of interest is the distribution of edge lengths, *σ*(*D*), which is defined by

$$\begin{array}{c} \int_{a}^{b}\sigma \left(D\right)dD=\text{Pr}\left(D_{ij}\in \left(a,b\right)\;|\;i\;\text{and}\;j\;\text{are}\;\text{connected}\right), \end{array}$$ (6)

where the right-hand side denotes the probability that *D*_*ij*_ is in the interval (*a*, *b*), conditioned on nodes *i* and *j* being connected by an edge. Note that

$$\begin{array}{c} \begin{array}{ccc} \text{Pr}(D_{ij} & \in & \left(a,b)\;\text{and}\;i\;\text{and}\;j\;\text{are}\;\text{connected}\right)\\ & = & P\int_{a}^{b}\sigma \left(D\right)dD\\ & = & \int_{a}^{b}p\left(D\right)\varrho \left(D\right)dD. \end{array} \end{array}$$ (7)

Since *a* and *b* are arbitrary, this implies the following instance of Bayes’ Theorem:

$$\begin{array}{c} \begin{array}{cc} \sigma (D) & =\frac{p(D)\varrho (D)}{P}\\ & =\frac{p(D)\varrho (D)}{\int_{0}^{D_{\text{max}}}p\left(D^{\prime }\right)\varrho \left(D^{\prime }\right)dD^{\prime }}. \end{array} \end{array}$$ (8)

It follows from (8) that

$$\begin{array}{c} \int_{0}^{D_{\text{max}}}\sigma \left(D\right)dD=1 \end{array}$$ (9)

as required. The probability density functions *σ*(*D*) and *ϱ*(*D*) and mean node degree *μ* can be measured from tomograms or taken from the literature, so we treat them as known and use them to determine *p*(*D*). From (8),

$$\begin{array}{c} p\left(D\right)=P\frac{\sigma (D)}{\varrho (D)}, \end{array}$$ (10)

and we can use (4) to express *P* in terms of *μ*. Since *N* is large, we replace (*N* − 1) by *N* in (4). Thus

$$\begin{array}{c} p\left(D\right)=\frac{\mu }{N}\frac{\sigma (D)}{\varrho (D)}. \end{array}$$ (11)

Eq (11) is a recipe for *p*(*D*) that will produce a random graph with the prescribed edge-length probability density function *σ*(*D*) and mean node degree *μ*. In order to be a valid probability, *p*(*D*) must satisfy 0 ≤ *p*(*D*) ≤ 1 for all *D*. Two conditions necessary for this to be true are that σ(D)=O(D) as *D* → 0 and *N* ≥ *μ* max_*D*_ (*σ*(*D*)/*ϱ*(*D*)).

Since the maximum edge length *D*_max_ is typically much smaller than the surface’s radius of curvature, we can neglect curvature so that to good approximation,

$$\begin{array}{c} \varrho (D)=2\pi D/S, \end{array}$$ (12)

where *S* is the surface area as stated above. Substituting this formula into (11) gives

$$\begin{array}{c} p\left(D\right)=\frac{\mu }{2\pi }\frac{\sigma (D)}{D}\frac{S}{N}. \end{array}$$ (13)

**Fig 1:**
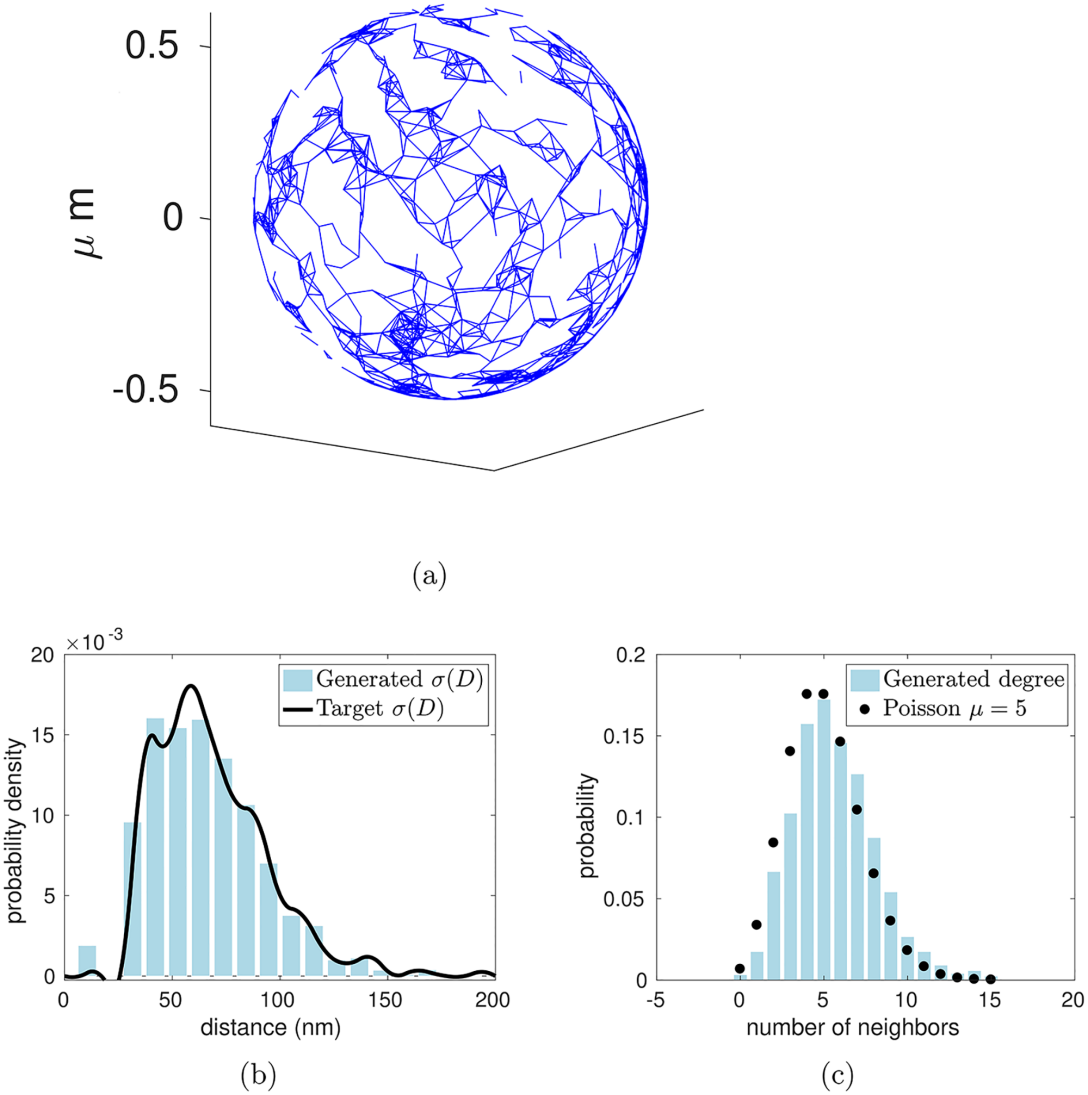
Random graph with specified statistical properties generated on the sphere. (a) Random graph on a spherical surface generated with a specified node density *N*/*S* = 300/*μ*m^2^ mean node degree *μ* = 5, and edge length distribution *σ*(*D*). Note that, while the density of nodes is consistent with the value for a whole red cell, the surface area of the sphere used in this test is a small fraction of the total surface area of a red cell. (b) and (c) Histograms demonstrating that the generated graph has the specified edge-length distribution *σ*(*D*) and Poisson node degree distribution, respectively.

As a validation test, we have used the random graph algorithm to generate a graph on the surface of a sphere with *N* = 1,200 nodes and mean node degree *μ* = 5, and setting the sphere radius so that the node density satisfies *N*/*S* = 300/*μ*m^2^. The node density is chosen to be in line with the observed density of junctional complexes in the red cell cytoskeleton, but note that the total surface area of the sphere used in this test is a small fraction of the surface area of an intact red cell. As shown in Fig 1, the statistical properties of the resulting random graph agree with the target distributions.

Note that in the case of the red cell cytoskeleton, the nodes are located in a thin layer below the membrane rather than on a true surface. This gives a correction to (12) that is described in the section “Density through slab” of S1 Text. However, since the formula (11) is valid in either case and given the continuous transition between surface and thin slab geometries we do not draw a significant distinction between the two cases here.

### Elastic response of random graphs

The cytoskeleton may for many purposes be considered as a continuum neo-Hookean material with a shear modulus *E* satisfying *E* ≈ 2 × 10^−3^ to 6 × 10^−3^ dyn/cm, as established through experiments and model studies [9]. Here, we use a simple two-dimensional test problem to compare this continuum formulation, discussed in detail in [1], to the discrete cytoskeleton model proposed in this article. We show that the energetic response to shear deformations of our discrete cytoskeleton model is in excellent agreement with a neo-Hookean material having a shear modulus in the experimentally determined range.

**Fig 2:**
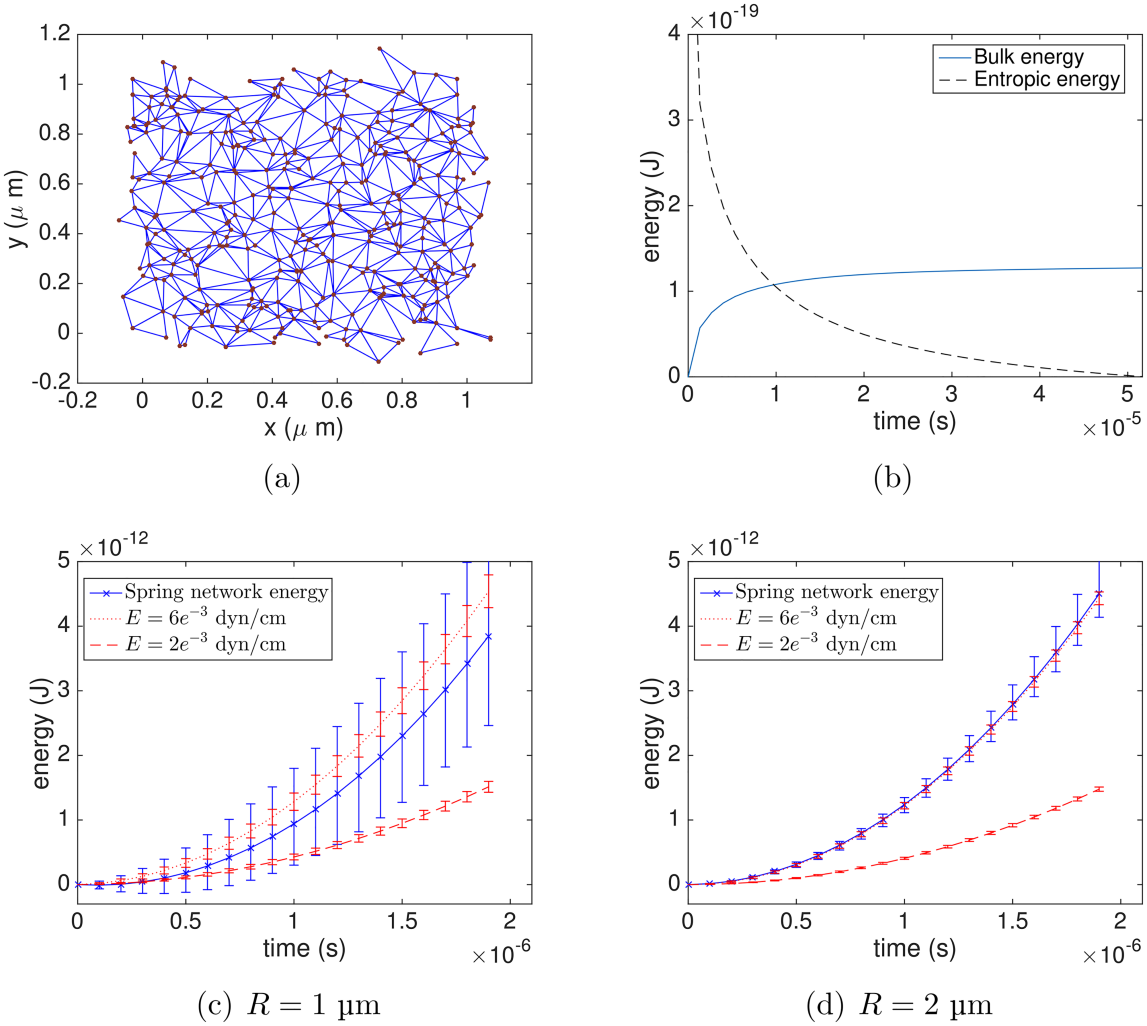
Comparison between the continuum shear energy and the energy of a random network of worm-like chains. (a) The triangulation associated with the random polymer network, (b) Relaxation to equilibrium of random network, (c)-(d) Comparison of energies from continuum shear formulation and random spring network on two periodic patches with *R* = 1 μm and *R* = 2 μm, respectively. The red curves are the corresponding energies computed from the continuum model using upper and lower values of measured shear moduli *E* obtained from the literature [9]. Error bars are computed by running simulations with several randomly generated networks.

We test several randomly generated model networks connected to a 2D sheet that resists changes in area. The random graph algorithm is used to generate a model cytoskeleton with mean node degree *μ* = 5, node density *N*/*S* = 300/*μ*m^2^, and edge-length probability density function (PDF) *σ*(*D*) on a periodic patch of membrane. The formulas used for *σ*(*D*) and *ϱ*(*D*) are computed from electron microscopy data, as will be described in the section “Data analysis”. Further, we compute a triangulation of the plane associated with the random graph by performing a Delaunay triangulation on the nodes (see Fig 2(a)).

This associated triangulation serves a dual role; it is used to enforce local area incompressibility, thus preventing the entropic spring network from collapsing, and to compute the continuum shear energy. To account for the periodicity, we surround the unit cell with periodic copies, triangulate the whole region, and keep the set of unique triangles that contain at least one point from the unit cell.

There are two contributions to the total elastic energy. First, each edge of the model cytoskeleton represents a worm-like chain, which is a model of an entropic spring with restoring force **F**_wlc_ given by Eq (4) of S1 Text. The total entropic force on a node with position **X** is

$$\begin{array}{c} \sum_{j}F_{\text{wlc}}\left(X_{j}-X\right), \end{array}$$ (14)

where ${\left\{X_{j}\right\}}_{j=1}^{n}$ is the set of *n* nodes connected by an edge to **X**. Second, there is a continuum bulk force **F**_bulk_(**X**) that penalizes changes in local area in the elastic sheet. The bulk energy is defined in terms of the reference configuration **Z** and the deformed state **X** via

$$\begin{array}{c} W_{\text{bulk}}=\frac{\kappa }{2}\int_{Z}{\left(J-1\right)}^{2}\text{d}a, \end{array}$$ (15)

where *κ* is the membrane bulk modulus, *J* is the Jacobian relating areas in the reference and deformed configurations, and *da* is the area element in the reference frame. By using the same nodes for the discretizations of both the membrane and cytoskeleton, so that both lie in the same plane with no slip allowed between them, we have made the idealization of strong vertical interactions between the membrane and cytoskeleton. In reality, the cytoskeleton and membrane can move relative to one another, and their relative deformabilities become evident in experiments such as micropipette aspiration in which the lipid bilayer becomes uniformly distributed in the tip of the pipette, whereas the cytoskeleton dilates and develops protein gradients in the tip [19]. The idealization of strong vertical connections is useful since it allows us to isolate the effects of changes to horizontal connections within the cytoskeleton that occur in hereditary elliptocytosis. Further, one of the constituents that make up the junctional complex and connects the cytoskeleton to the lipid membrane, the transmembrane protein Band 3, has a long-range diffusion timescale on the order of seconds [19, 30]. Therefore, holding it fixed to a certain point in the membrane is reasonable for our simulations, which take place over a few hundredths of seconds and involve network remodeling timescales on the order of 0.01–0.1 seconds. Note that the cytoskeleton resides in a thin layer with a width of just 200 nm beneath the membrane, as observed for instance in the tomograms analyzed here. Since this thickness is small compared to the red cell diameter, it is reasonable to make the approximation that the membrane and cytoskeleton occupy the same surface.

Since we consider the red cell membrane to be locally area-incompressible, in practice *κ* is a penalty parameter that is set such that the absolute value of local changes in area are not greater than 2–4% on average [31]. In our simulations, we use values for *κ* in the range 0.1–1.0 dyn/cm. We have neglected the cytoskeleton bulk modulus since it is smaller than the membrane bulk modulus by several orders of magnitude [32]. On a triangulated domain, *W*_bulk_ can be as discretized as in [1] with

$$\begin{array}{c} W_{\text{bulk}}=\frac{\kappa }{2}\sum_{s}{\left(\frac{\text{area}\left(t\right)}{\text{area}\left(s\right)}-1\right)}^{2}\text{area}\left(s\right), \end{array}$$ (16)

where *s* and *t* are triangle indices in the reference and deformed configurations, respectively. The force at node **X** due to bulk elasticity is given by **F**_bulk_(**X**) = −∇_**X**_ *W*_bulk_. The total force is simply the sum of entropic and bulk elasticity forces.

We fix the time step Δ*t* = 1 ⋅ 10^−9^ s and *κ* = 0.2 dyn/cm, and move the nodes by overdamped dynamics $\zeta \dot{X}=F\left(X\right)=F_{\text{bulk}}\left(X\right)+\sum_{j}F_{\text{wlc}}\left(X_{j}-X\right)$ using the forward Euler method. In a pre-computation, we let the nodes equilibrate as shown in the energy curves of Fig 2(b). The friction coefficient is calculated through the Stokes relation *ζ* = 6*πνr* with a radius of 15 nm for the actin nodes [5] and dynamic viscosity *ν* = 1.2 cP, resulting in a value of *ζ* ≈ 3.3 ⋅ 10^−7^ g/s.

Next, the nodes are advected in the prescribed incompressible flow

$$\begin{array}{c} u=(\text{cos}\;2\pi y/R,\text{sin}\;2\pi x/R), \end{array}$$ (17)

and changes in the entropic spring energy and continuum shear energy are observed over a 2 μs timespan. This test is performed using square unit domains of length *R* = 1 μm and *R* = 2 μm with periodic boundary conditions, repeating 10 times on each domain with different randomly generated networks and discarding any trials in which the mesh becomes tangled. On the timescale over which this deformation is applied, the nodes of the network undergo a maximum displacement of 14% relative to the contour length *L*. Although the displacements from the initial configuration are small, the initial edge lengths themselves are not necessarily small (based on the tomogram data), and we find that the maximum strain ‖**r**‖/*L* experienced by edges is 93%. Although the mean strain is significantly smaller, nonlinearities in the polymer force do therefore play a role.

We find that the behavior of the random entropic spring network falls within the experimental range of the continuum shear energy, as illustrated in Fig 2(c)–2(d). Note that, since the deformation is prescribed, the shear energy could be computed analytically. However, we find it convenient to approximate using a discretization on triangles, as done later on to calculate bulk energies in the 3D simulations. Although the continuum shear energy changes deterministically on the elastic sheet, the error bars over the red curves in Fig 2(c)–2(d) come from calculating it on several different random triangulations.

These tests show that the total entropic spring energy increases remarkably like the continuum shear energy, and that the discrete cytoskeletal network has an effective shear modulus within the experimentally determined range. Performing this simulation using several random networks, we find that the variance in the computed energies decreases as the domain gets larger. This is expected since the random networks generated appear quite different when viewed close-up, but are alike on larger scales since they all have identical statistical properties. These results demonstrate that, because of the cytoskeleton’s locally irregular structure, the variance in measurements of its shear modulus depends on the scale of observation.

### Tomogram preparation

**Fig 3:**
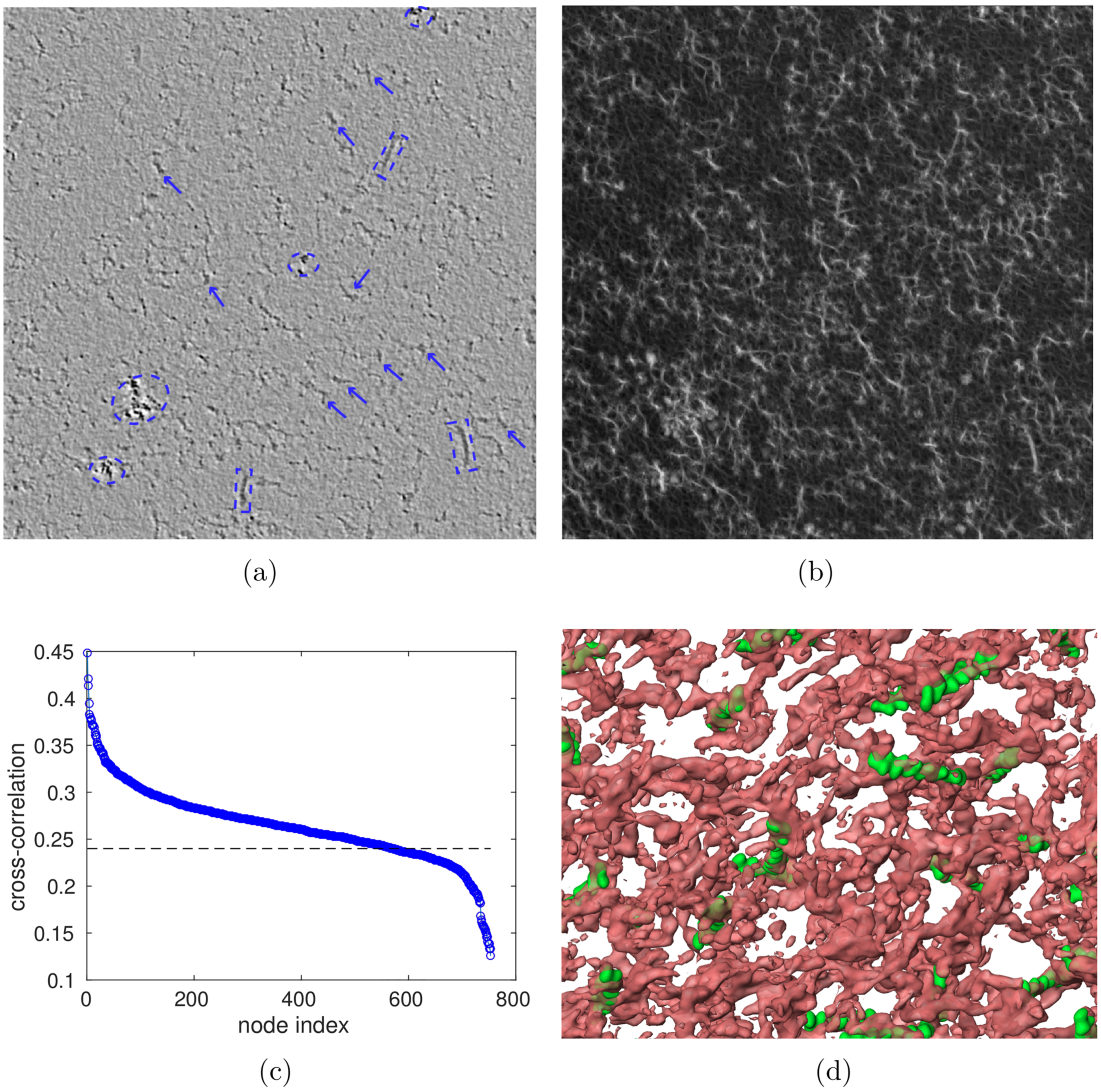
Cryoelectron tomography and pattern matching of RBC skeletons. (a) A virtual slice of a cryoelectron tomogram of a red blood cell skeleton. Putative junctional complexes containing the actin protofilaments are indicated with arrows. Structures inside dashed lines are uncharacterized protein clusters (circles) and lipidic remains (squares), (b) Cross-correlation map resulting from comparing the actin template with the tomogram. The brighter the spot, the higher the probability of a good match, (c) The corresponding cross-correlation coefficient plot with a line indicating the cutoff value used. The 2^nd^ knee of the curve is used to avoid false negatives, (d) The identified actin protofilaments overlaid on the actual densities of the tomogram with obvious false positives discarded (for example, some spots with high cross-correlation values are found at locations of lipid remains and unknown protein clusters).

The edge-length PDF *σ*(*D*) and node-node distance PDF *ϱ*(*D*) used in the random graph algorithm are based on three-dimensional images of the isolated red cell cytoskeleton obtained by cryoelectron tomography (Fig 3(a)). The use of such image data ensures that our model cytoskeletons have realistic statistical properties. Methods for separating and preparing the cytoskeletons for electron microscopy are described in [5]. The tomographic images reveal the convoluted structure and irregular topology of the native cytoskeleton, in contrast to some early cytoskeleton models mentioned in the introduction that assumed the topology of the cytoskeleton to be that of a hexagonal lattice. Although such a hexagonal topology is suggested by electron microscopy images of the spread and negatively stained cytoskeleton, it has been suggested [5, 33] that this topology results from negative staining and/or network reorganization in response to stretching and adsorption to a carbon substrate. To be able to reproduce the observed irregularity with our random graph model, we first analyze tomographic images to gather statistics on the network topology, contour lengths, and end-to-end distances of putative spectrin tetramers. Since the tomograms do not distinguish between different protein species, in order to extract these distributions from the three-dimensional images, a segmentation algorithm must be used to identify the cytoskeleton constituents.

### Image processing

Our segmentation algorithm to extract the statistical data for our model consists of two steps. In the first step, actin polymers are identified within the tomogram using an existing method [34] based on correlation with the spatial electron density of a 13 subunit-long filament of actin (Fig 3). We use the software package MolMatch [34], which rotates the electron density computed from the known crystal structure of actin through various Euler angles and computes the correlation at each voxel of the tomogram. The positions with the highest correlations are designated as junctional complexes, subject to the constraint that no two nodes are closer than 14 nm. Although the actin polymers take up a non-trivial volume relative to the cytoskeleton, they are considered to be points for the next step of the segmentation algorithm.

In the second step, the tomogram is converted to a three-dimensional binary image by choosing a density threshold and setting all values above and below the threshold to be 1 and 0, respectively. (In what follows, the cytoskeleton refers to the set of voxels with value 1.) The threshold is chosen in order to produce a mean number of edges per node of *μ* = 5, in accordance with published values based on direct inspection of cytoskeletons [5, 33]. Next, the largest connected component of the cytoskeleton is found by using flooding, i.e. breadth-first search.

**Fig 4:**
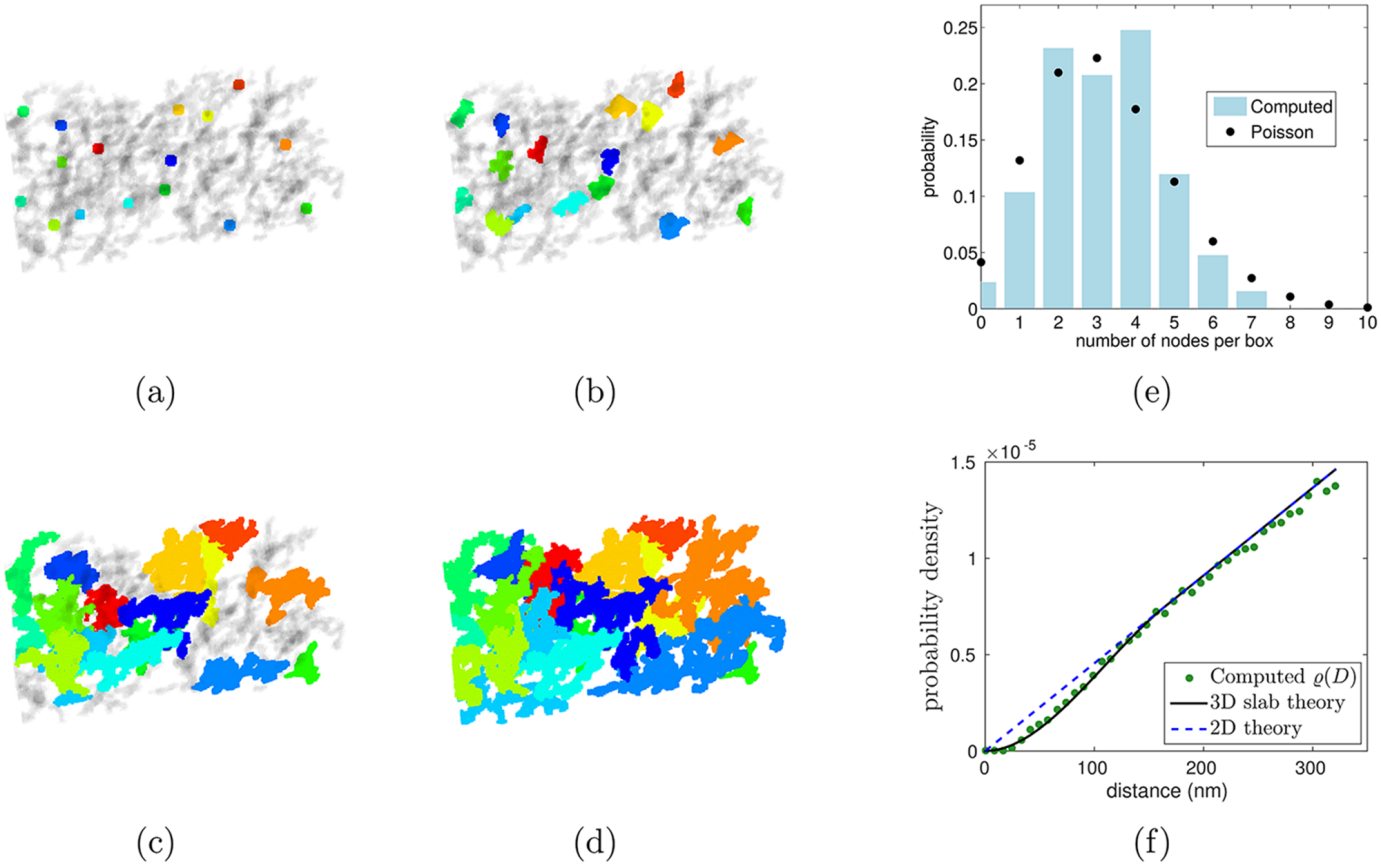
Image processing procedure. (a)-(d) The centers of mass of the actin protofilaments shown in Fig 3(d) are used as nodes for the watershed procedure that segments the cytoskeleton. This segments the cytoskeleton into regions associated with the nodes, as illustrated here with different colors. (e) Histogram of nodes per box compared to the Poisson distribution, (f) Plot of observed pairwise distances compared to the density PDF for uniformly distributed points in a 3D slab.

Restricting attention to nodes within this largest connected component, we next segment the skeleton using a watershed algorithm [35] that, in addition to computing the topology, yields geometrical information about the end-to-end distances and contour lengths of spectrin tetramers. Though similar to a standard breadth-first search, the watershed algorithm is different in that instances of flooding are launched synchronously from each node, stopping in voxels where instances initiated from different nodes meet. The measure of distance implicit in this segmentation is *not* the Euclidean distance, but rather the shortest path length through the connected component, as approximated by counting the number of steps through adjacent (non-diagonal) voxels. Interpreting this procedure in terms of the cytoskeleton structure, the halfway points at which instances of flooding meet are the locations at which spectrin dimers join to form tetramers. These halfway points are also located near the binding sites of the protein ankyrin, which links the cytoskeleton to the red cell membrane. See S1 Video and Fig 4(a)–4(d) for illustrations.

## Results

### Data analysis

Our first goal is to extract parameters relevant for our simulations from the 3D images produced by electron tomography. In particular, we use our segmentation algorithm to determine the edge-length PDF *σ*(*D*) and node-node distance PDF *ϱ*(*D*), and test whether these distributions are consistent with the assumptions of the random graph algorithm and entropic spring model, respectively.

We use two tests to establish that the nodes in the tomogram satisfy the assumption in the section “Generating the cytoskeleton” that the nodes are uniformly distributed. For the first test, we partition the tomogram (with approximate dimensions 400 nm × 900 nm × 160 nm) into 125 boxes of approximate size 80 nm × 180 nm × 32 nm. If the nodes are uniformly distributed, the probability that a given node lies within a particular box will be *r* = 1/125 = 0.008, i.e. the ratio of the box’s volume to the volume of the entire tomogram. More generally, for *N* total nodes, the probability that *k* nodes lie within the same box is given by the binomial distribution, which may be approximated as Poisson with mean *rN* since *r* is small and *N* is large. Fig 4(e) shows that the histogram of nodes per box is indeed in close agreement with the Poisson distribution.

**Fig 5:**
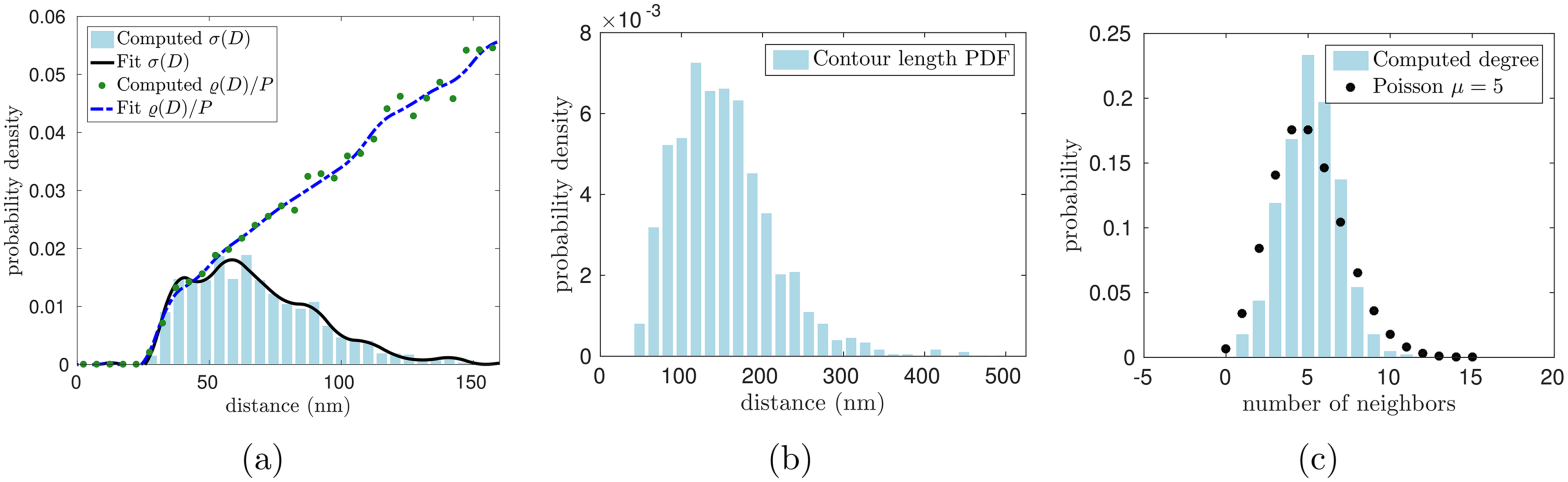
Results of the segmentation algorithm. (a)-(c) Probability density functions of edge-lengths versus distances between nodes, contour lengths, and probability mass function of the number of connections per node computed from tomograms by our segmentation algorithm.

For the second test, *ϱ*(*D*) is extracted from the tomogram and compared to an analytic formula for uniformly distributed nodes derived in the section “Density through slab” of S1 Text (see Fig 4(f)). To extract *ϱ*(*D*) from the tomogram (see Fig 5(a)), we compute the distance *D*_*ij*_ between each pair of nodes *i* and *j* and create a histogram on the interval [0, 300] nm. Edge effects have been mitigated by placing periodic copies in the horizontal directions, and only counting those pairs with at least one member in the original volume. To correctly normalize this distribution, we compute the vertically-averaged volume of intersection *V*_*I*_(*η*, *D*_max_) between the slab of thickness *η* = 160nm and a sphere of radius *D*_max_. This computation is similar to those in the section “Density through slab” of S1 Text and we only state the result here: $V_{I}\left(\eta ,D_{\text{max}}\right)=\pi D_{\text{max}}^{2}\eta -\pi \eta^{3}/3$ in the case of interest that *D*_max_ > *η*. The resulting distribution is consistent with the presence of uniformly distributed nodes in the skeleton, as shown by the close match to the analytical formula computed in the case of uniformly distributed points in a 3D slab.

To extract *σ*(*D*) from data, we make a histogram of the end-to-end distances between nodes identified as neighbors by the segmentation algorithm (see Fig 5(a))). We observe that the computed *σ*(*D*) vanishes below a certain cutoff value. The template-matching algorithm imposes a lower cutoff of 14 nm to prevent multiple identifications of the same junctional complex, but in fact the edge length distribution that arises from the segmentation algorithm reveals very few nodes connected by distances of less than 30 nm. This is consistent with the physical characteristics of the cytoskeleton: since the junctional complexes themselves have a radius of about 15 nm [5], excluded volume prevents their centers from coming within 30 nm of one other.

The contour-length PDF and probability mass function of connections per node are computed similarly (Fig 5(b)–5(c))). We find that the distributions extracted by the segmentation algorithm are consistent with the constraint *σ*(*D*) ≤ *ϱ*(*D*)/*P* on inputs to the random graph model described in the section “Generating the cytoskeleton”. Recalling that *P* is the probability that two nodes are connected, that *μ* is the average number of connections at each node, and that *N* is the total number of nodes, *P* = *μ*/*N* under the assumption that all connections are independent. This assumption is justified by the data because, according to Fig 5(c), the observed node degree distribution is close to being Poisson. Since *μ* = 5 by design and the total number of junctional complexes satisfies *N* ≈ 40,000 in red cells [36], the value of *P* is fixed. Therefore, the inequality *σ*(*D*) ≤ *ϱ*(*D*)/*P* becomes a constraint on the PDF’s *ϱ*(*D*) and *σ*(*D*). Fig 5(a) shows that the extracted distributions satisfy this constraint to within experimental noise (i.e. the blue histogram lies nearly beneath the green dots).

The template-matching algorithm for identifying junctional complexes results in a density of approximately 340 points/*μ*m^2^, which is in reasonable agreement with the experimentally-determined density of 290 points/*μ*m^2^ computed using a total of 40,000 junctional complexes [36] and the surface area 138 *μ*m^2^ [9]. However, visual inspection reveals both false positives and false negatives. It would be valuable to validate the template-matching algorithm experimentally, for instance by labeling actin or ankyrin and using super resolution fluorescence microscopy.

### Generating the model cytoskeleton on a triangulated surface

In order to carry out simulations on whole red cells, we used the random graph algorithm of the section “Generating the cytoskeleton” to generate a full model cytoskeleton on a triangulated surface representing a whole cell using the distributions *σ*(*D*) and *ϱ*(*D*) extracted from data together with the target density of 290 nodes/*μ*m^2^. The number of random points on each triangle is drawn from a Poisson distribution. Each of these points is given a random position that is chosen independently from the uniform distribution on the corresponding triangle. This is done by generating candidate points within the bounding rectangle of the triangle and then rejecting points that fall outside the triangle. The end result of the above construction is that we have distributed the nodes according to a Poisson process on the whole triangulated surface with the target density (see the section “Immersed boundary method” in S1 Text for a close-up of the nodes and triangulation).

For the subsequent step of determining which pairs of nodes are connected by entropic springs, the large number of nodes makes it impractical to test each pair explicitly. Instead, we bin the nodes into *N*_box_ boxes of edge length at least *D*_max_ in each direction. This makes the determination of edges more efficient, since for a given node only the approximately $O(N/N_{\text{box}})$ nodes in the same or adjacent boxes must be tested as candidate neighbors. We find that the resulting graph is percolated; over 99% of the total nodes belong to the same connected component.

Note that the cytoskeleton tends to stay attached to the membrane in our simulations since all points move in the same interpolated velocity according to the immersed boundary formulation (Eq (16) of S1 Text). In the absence of a membrane, the model cytoskeleton is compressible; however, in our simulations the cytoskeleton moves in the same velocity field as the incompressible membrane. This is analogous to considering the motion of tracer particles within an incompressible fluid; although the tracer particles themselves do not resist compression, their local density does not change over time by virtue of the incompressibility of the fluid.

### Simulating response to flow and applied strain

In order to test the response of our model skeleton to flow, we examined the response of the red cell to different flow conditions using the immersed boundary method (see the section “Immersed boundary method” in S1 Text). The model cytoskeleton resists in-plane shear deformation, whereas the lipid bilayer resists bending and changes in local area. We simulate a red cell with equal internal and external fluid viscosities (i.e. a red cell ghost) and examine how the edge length distribution changes during the resulting motion. A shear flow is generated by applying equal and opposite body forces in two planes of the computational domain, as in [1, 37]. The strength of the resulting flow is given in terms of the dimensionless capillary number *G*, defined by $G=\mu \dot{\gamma }a/E$, where *μ* is the dynamic viscosity, $\dot{\gamma }$ is the shear rate, *a* is the effective cell radius, and *E* is the shear modulus as above.

Placing cells in shear flow produces tank-treading behavior, in which cells elongate and align their long axis toward the flow, with the membrane revolving around the perimeter of the cell in a periodic fashion [38, 39]. This complex behavior is a good test of the model because tank-treading frequencies can be quantified and compared with existing values in the literature. This test not only helps validate the cytoskeleton model: it can also be used to demonstrate the effect of network dynamics on a cell’s response in flow.

**Fig 6:**
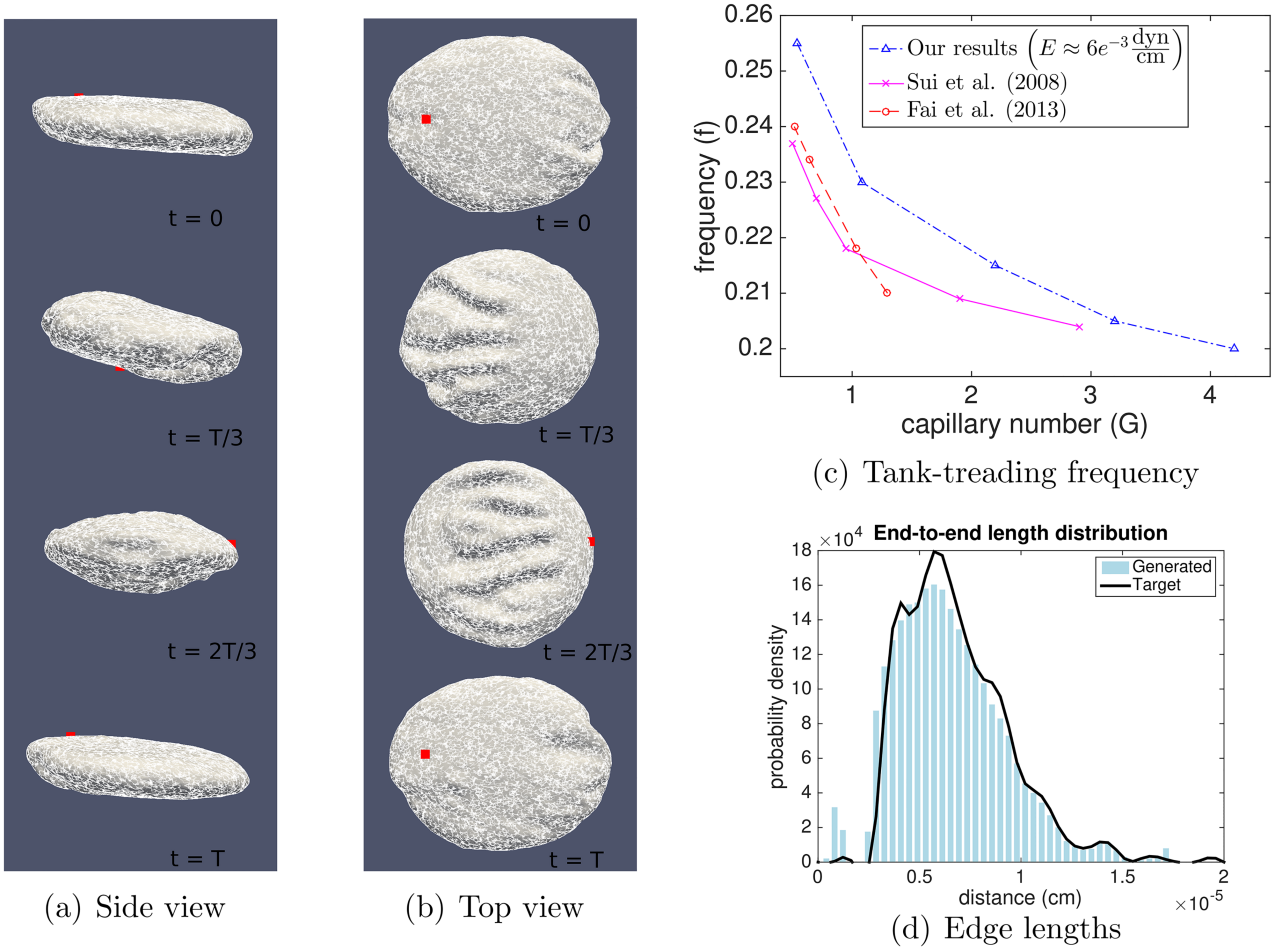
Red cell ghost in steady shear flow with fixed cytoskeletal topology. See also S2–S4 Videos. (a) Side view of a red cell tank-treading in shear flow with capillary number *G* = 0.54 and period *T* = 0.026 seconds. Based on the results of the section “Elastic response of random graphs”, the capillary number is calculated using the shear modulus *E* = 6 × 10^−3^ dyn/cm. A material point is marked in red to illustrate the counterclockwise rotation of the cell membrane, (b) Top view of the same cell, (c) Frequency of tank-treading versus capillary number for several values of *G* and comparison to previous results [1, 41], (d) The distribution of edge lengths in the cytoskeletal network is observed to oscillate during tank-treading.

In the flow regime we investigate, red cell ghosts undergo a *breathing* motion that is intermediate between tumbling and tank-treading, the behavior seen at high shear rates. We find the dependence of the nondimensional frequency $f=2\pi /(T\dot{\gamma })$ on the breathing period *T* computed in our simulations to be consistent with previous studies [1, 40] (see Fig 6(c)). Over the course of the cell’s breathing motion, we monitor the edge length distribution of its cytoskeleton (see Fig 6(d) and S2–S4 Videos). S4 Video shows that the edge length distribution oscillates with each breathing period.

In contrast to the above simulations in which the network connectivity has been taken to be static, there is experimental evidence that the cytoskeleton continually remodels over time. The rate of remodeling is not yet well-characterized; Ungewickell and Gratzer report that the timescale is of the order of 10 minutes for a red cell at rest [42] and Fischer reports a stress relaxation timescale ≳ 10 hours [43], while others have reported a more rapid, highly shear-dependent remodeling in red cell ghosts with significant implications for the red cell’s deformability [7, 44]. It has been suggested that hemoglobin may stabilize the cytoskeleton, which could explain the faster remodeling observed in red cell ghosts [43], but there is no consensus in the literature on the reason for the discrepancy in remodeling timescales. To examine the hypothesis that network dynamics plays a key mechanical role, we test the effect of network dynamics on our model by incorporating rate constants *k*_on_ and *k*_off_ for edge formation and breakage, respectively. We model the network dynamics in a stochastic manner using an on-rate that is length-dependent and an off-rate that is independent of length (see the section “Dynamic connections” in S1 Text and S5 Video, a close-up of the remodeling cytoskeleton in a cell at rest).

**Fig 7:**
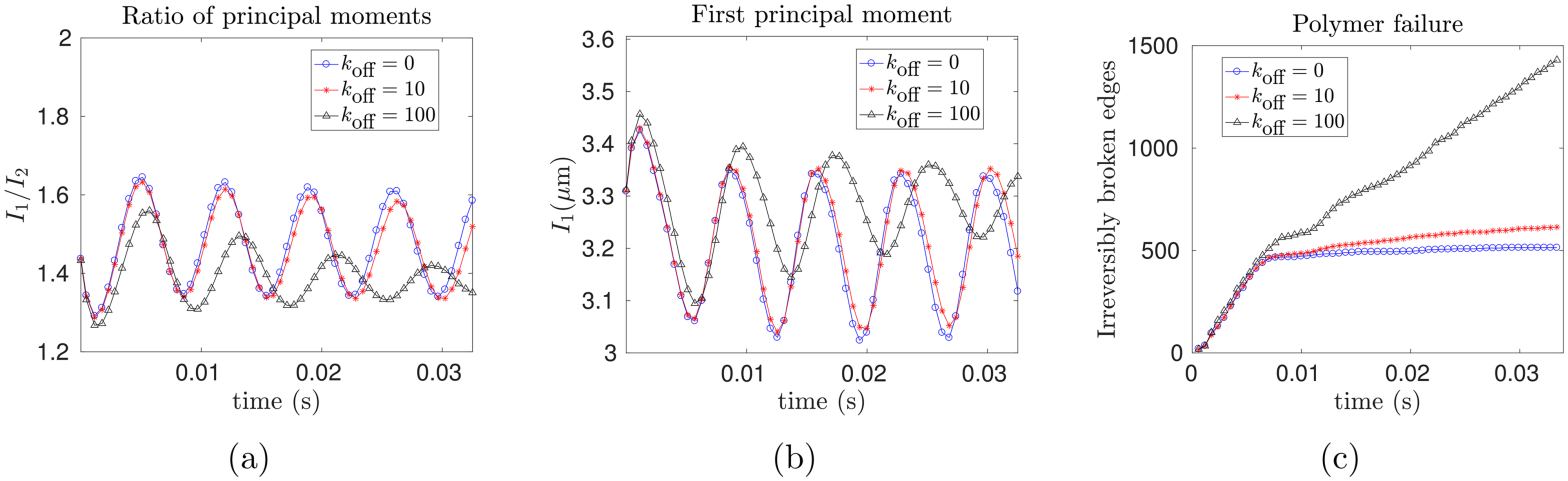
Behavior of a dynamic cytoskeleton under shear flow. (a) *I*_2_/*I*_1_ over time for *k*_off_ = 0, 10, and 100 s^−1^, (b) The lower effective shear modulus for a dynamic network leads to a higher dimensionless capillary number and greater deformations, as evidenced by the greater maximum value of *I*_1_ for the network with the fastest rate of remodeling, (c) The total number of irreversibly broken edges is plotted versus time. The network with the fastest dynamics, i.e. *k*_off_ = 100s^−1^, accumulates the most irreversibly broken edges in shear flow. The benefit of having more edges that spontaneously disconnect before breaking is outweighed by the cost of decreased shear resistance and greater extension.

We repeat the shear flow simulations, now including network dynamics, and observe the changes in the cytoskeletal structure over time. In order to follow shape changes, we define *I*_1_ ≥ *I*_2_ ≥ *I*_3_ ≥ 0 to be the ordered eigenvalues of the moment of inertia tensor of the red cell membrane. The moments of inertia are related to the principal axes of an ellipsoid, so that changes in the ratio *I*_1_/*I*_2_ correspond to shape deformations. Fig 7(a) shows increasing tank-treading periods and overall deformations as *k*_off_ increases from *k*_off_ = 0, 10, and 100 s^−1^, which we interpret as a loss of elasticity and an increase in viscoelastic creep as the network becomes more dynamic. Fig 7(a) shows the breathing period to be ≈0.02 s, and in our simulations the network dynamics are observed to have a significant effect when the timescale of remodeling $k_{\text{off}}^{-1}$ is of the same order, i.e. *k*_off_ ≈ 100s^−1^.

One potential physiological advantage of having dynamic network connectivity is that it may decrease the chance of polymer failure. To model this behavior, we assume that a bond is broken irreversibly when its length exceeds *L* = 200 nm, which is the unfolded contour length of spectrin [45]. This failure model can be interpreted biologically as a spectrin tetramer unfolding upon being sufficiently stretched, so that it no longer acts like a spring, with refolding requiring times so long that it may be neglected over the course of the simulation. Experimental evidence for spectrin unfolding under extension has been presented in [46–48] and ankyrin has also been shown to unfold in response to large forces [49]). Upon incorporating polymer failure in this manner, we next ask: do network dynamics decrease the number of irreversibly broken bonds over time?

Somewhat counterintuitively, we find that the presence of network dynamics in shear flow *increases* the number of edges passing the threshold for irreversible breakage, as shown in Fig 7(c). The explanation for this observation is that network dynamics makes the cell less elastic, decreasing the shear modulus *E* and consequently increasing the dimensionless capillary number, which is inversely proportional to *E*. The benefit of having edges that spontaneously disconnect before the threshold is reached is outweighed by the cost of decreased shear resistance and greater extension seen by plotting the cell’s first principal moment of inertia (Fig 7(b)).

**Fig 8:**
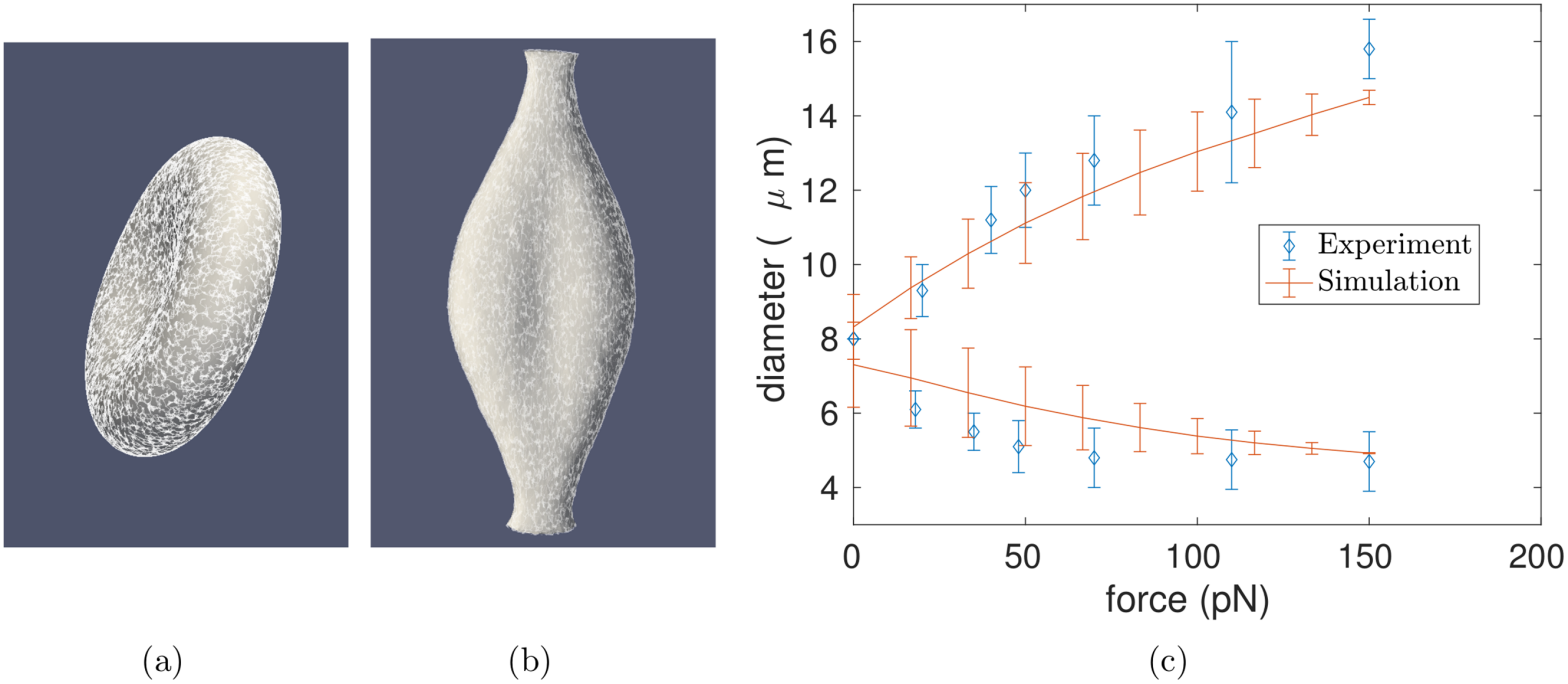
Mechanical properties of red cell model with static spectrin networks under deformation typical of an optical tweezer experiment. (a) Initial shape of membrane with overlaid cytoskeleton, (b) Final state (see also S6 Video), (c) Comparison of the simulated force-extension curve to optical tweezer experiments [53], in the axial (upper curve) and transverse (lower curve) directions. Error bars from the simulation were computed by comparing forward and reverse deformations.

To isolate the effect of network dynamics from that of extension in shear flow, we considered a situation in which we prescribe the strain, rather than the shear stress, on the cell. Inspired by optical tweezer experiments and simulations [13, 50–53], the cell is attached by stiff springs at both ends to small clusters of virtual tether points. Of the approximately 40,000 vertices composing the triangulated mesh, about 1,500 vertices are attached to tether points. The tether points are uniformly distributed over 3–4% of the red cell surface area. The motion of the tether points is prescribed to pull the ends of the cell in opposite directions at a constant rate and place the cell under increasing tension. As noted above, prescribing the extension rate rather than the force allows us to isolate the effect of network dynamics from changes in overall shape. To help validate the model, we compare the force-extension curves obtained from simulation to the results of optical tweezers experiments [53]. The force-extension curves calculated using a static spectrin network are in agreement with experiment results (Fig 8(c)).

**Fig 9:**
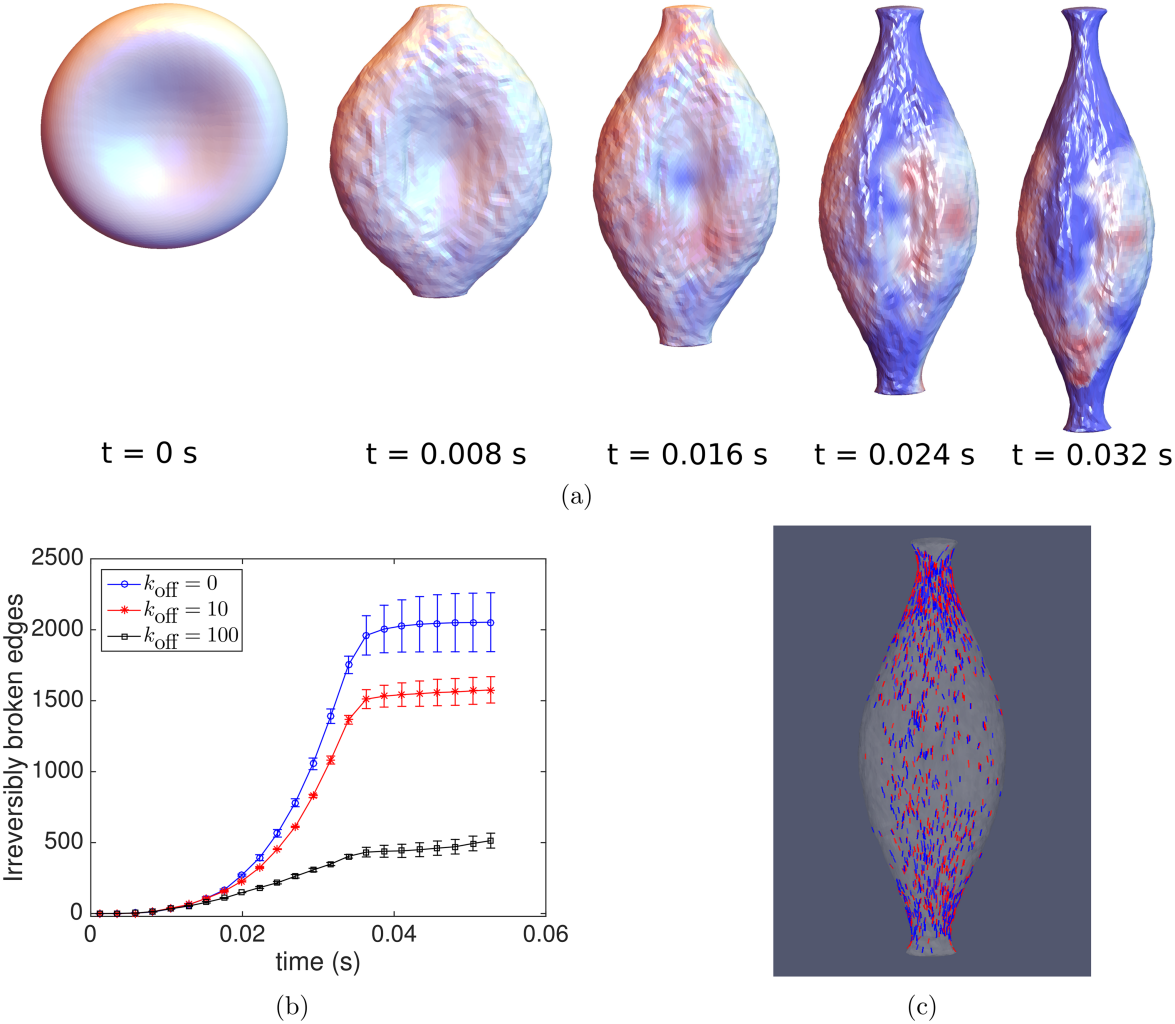
Comparison of static and dynamic spectrin networks under deformation typical of an optical tweezer experiment. (a) Heat map on the cell surface representing the number of irreversibly broken edges on the static (blue) and dynamic with *k*_off_ = 10s^−1^ (red) networks at several instants in time. The static network has an excess of irreversibly broken edges at the cell ends, as illustrated by the deep blue color, (b) Dynamic networks (*k*_off_ = 10s^−1^, red symbols and *k*_off_ = 100s^−1^, black symbols) accumulate fewer irreversibly broken edges under a prescribed strain than the static network (*k*_off_ = 0s^−1^, blue symbols). Error bars were computed by performing each simulation at 64 × 64 × 128 and 128 × 128 × 256 grid resolutions, (c) Final state of stretched membrane with irreversibly broken edges overlaid from dynamic network (red edges) and static network (blue edges) simulations.

In the following simulations, we replace the worm-like chain model on each edge of the spectrin network by a linear entropic spring force given by Eq (3) of S1 Text that has the same behavior at small strains. Although the linear spring force allows for infinite extension in principle, large extensions will not occur in our simulations since spectrin tetramers break irreversibly upon reaching their contour length. Further justification is provided in the section “Dynamic connections” of S1 Text. Upon extending the cell by about 100% over the course of approximately 0.23 seconds, we find that the total number of irreversible breakage events decreases by about 20% in the presence of network dynamics (Fig 9 and S6 Video). As shown in Fig 9, the cytoskeleton edges become less dense in regions of high strain where more irreversible damage occurs. Since the spectrin network initially has approximately 110,000 edges, the 2,000 edges broken over the course of our simulation make up less than 2% of the total network and have a negligible effect on the overall cell mechanics. However, the difference in breakage rates is greatly magnified over the course of time and in the context of positive feedback. Whereas we have considered only one full deformation cycle because of computational constraints, the average transit time through the circulation is approximately twenty seconds, so that a red cell experiences on the order of 10^5^ such cycles over their lifespans [32]. These deformations can be extreme, e.g. when passing across the spleen’s narrow endothelial slits having dimensions of approximately 2 μm ×1 μm [54, 55]. There is positive feedback because the more broken bonds a cell has, the less able it is to return to its rest shape after large deformations, and therefore the more likely it is that remaining bonds will become progressively stretched and break.

It is surprising that, depending on the type of deformation the red cell is subjected to, the presence of dynamics can either lead to more or less damage to the cytoskeleton, but it is consistent with previous studies showing that dynamics can generate both enhanced and deficient spectrin networks [17]. This prediction of how cytoskeletal dynamics decrease the number of polymer failures over time could be tested experimentally by using optical tweezers to deform red cell mutants in which the persistence of spectrin tetramer connections has been altered. For example, hyperstable spectrin tetramers that disconnect less frequently than wild type cells and have been produced in transgenic mice (N. Mohandas, New York Blood Center, personal communication, 2015).

## Discussion

We have used our image-based model to investigate the physiological consequences of certain cytoskeletal properties at microscopic scales, specifically the importance of network dynamics. We have used simulations to address the consequences of allowing the spectrin network to reorganize over time, an effect which is thought to take place *in vivo*. This model predicts that, in the presence of cytoskeletal reorganization, repeated deformations will lead to changes in the structure of the cytoskeleton. When a cell undergoes tank-treading in shear flow, we find that faster cytoskeletal reorganization leads to more irreversibly broken spectrin tetramers and a smaller dimensionless tank-treading frequency. This is because the loss of elasticity from remodeling leads to larger capillary numbers, which causes greater extensions and has previously been shown to generate smaller dimensionless tank-treading frequencies [41, 56].

In contrast to the case of shear flow, results from our model suggest that, when the cell is placed under a repeated strains, cytoskeletal dynamics may play a protective role by allowing spectrin tetramers to disconnect before they would break. In particular, we found that by allowing transient disassembly of spectrin tetramers, the cytoskeleton suffered fewer irreversibly broken edges in response to applied strains that simulate the conditions of optical tweezer experiments. We used a relatively fast disassociation constant of at least *k*_off_ = 10s^−1^, based on the rapid remodeling that was previously reported [7, 44]. However, recent measurements suggest that the stress relaxation timescale is at least 10 hours [43], indicating that the true rate of remodeling is significantly slower than the value of *k*_off_ = 10s^−1^ used for our modeling. Any potential protective effect of dynamics may therefore only be significant on the timescale of hours to days, over which cells undergo hundreds to thousands of deformation cycles. The same results also suggest that, under conditions of fixed strain, mutant red cells with static connectivity may accumulate damage more quickly than dynamic wild type cells. Further study using biophysically-realistic models of networks dynamics, together with optimized algorithms and higher thoroughput simulation techniques, is needed to quantify these predictions over a wider range of parameter space. Empirical testing of this hypothesis by using optical tweezers or microfluidic devices [57] to apply strain or shear stress to red cells with hyperstable spectrin tetramers would be an important step in validating the model and identifying any possible protective effect of network dynamics on the cytoskeleton.

This model could be used to investigate in detail the consequences of mutations that occur in hereditary elliptocytosis, a genetic disorder that affects proteins responsible for horizontal connections within the spectrin cytoskeleton [58]. The statistical properties of the spectrin network are likely to be affected by these mutations. Our model makes it possible to investigate mechanisms of the disease, since we can define parameters that govern cytoskeletal structure, including the number of junctional complexes, the number of polymers attached to each junctional complex, the length of the polymers, the polymer elasticity, and network dynamics. The microscopic details that govern polymer connections in the cytoskeleton can have an effect on the macroscopic behavior, as evidenced by the change in the tank-treading frequency caused by dynamics within the spectrin network. As noted in the introduction, composite models have been developed [16–18] that capture the disruptions in vertical connections between the membrane and cytoskeleton that occur in hereditary spherocytosis and during extreme deformations. Although we have enforced strong vertical connections in the present work, it is possible to incorporate relative motion within the immersed boundary framework by including a slip velocity [59]. Given the recent progress on composite models this would be an interesting application of our image-based approach.

This approach starts with structural tomography data, uses a random graph algorithm to generate a representative cytoskeleton, and then simulates the behavior of a cell with those cytoskeletal properties under realistic flow conditions. In order to make this computational framework generally valuable for studying questions in red cell physiology, the existing limitations of this method must be addressed. With regard to the segmentation algorithm used for processing tomographic images, one challenge is to accurately identify the junctional complexes linked by spectrin. The method used, which involves cross-correlation to actin’s known electron density, is appealing because it gives reasonable results and can be done using freely available third-party software. However, visual inspection suggests both false positives and false negatives. Establishing the accuracy of this method, e.g. through experiments in which different cytoskeletal components are labeled by streptavidin [60], would be an important validation of the node identification step. Further, the threshold used to binarize the tomograms is presently determined by prescribing the mean degree *μ*, but it would be preferable if *μ* were an output out of the data analysis. We have not taken this approach since *μ* has been found to be sensitive to the threshold used, making it difficult to identify a value robust to the image processing parameters. It is possible that using a single threshold is too simplistic; by considering the known electron densities of spectrin and actin, it may be possible to compute more appropriate independent thresholds. Of course, tomograms with a higher signal-to-noise ratio would greatly aid our analysis and the new direct detectors currently being used for electron microscopy are likely to make this possible.

Several simplifying assumptions have been made in the cytoskeletal model presented here, including the independence of edges in the random graph model, the treatment of spectrin polymers as Hookean springs with no self-avoidance, and our particular implementation of spectrin network dynamics. Further investigation may reveal the need for more complicated models. For example, in addition to forming tetramers, spectrin dimers can join together in the red cell cytoskeleton to form hexamers and other higher-order oligomers. Although the number of such higher-order oligomers is significantly less than the number of tetramers, it has been shown that including even a relatively small number of hexamers can drastically change the network elasticity [22].

We note that spectrin cytoskeletons play important physiological roles in other settings as well: they have been shown to be important regulators in *Drosophila* development and to be present in axons, to which they may provide structural stability to help the axons span long distances [61, 62]. They also play a role in cardiomyocyte differentiation and heart development [63]. Dysfunction in the spectrin-dependent cytoskeleton in cardiomyocytes has been shown to underlie severe arrhythmia associated with aberrant calcium phenotypes, identifying spectrin as critical for normal myocyte electric activity [64]. Although we have focused here on spectrin networks, the modeling of networks made up of polymers besides spectrin is of course of significant interest. For example, Lee et al. [65] examined cytoskeletal remodeling in fibroblasts, in which the cytoskeleton is made up of actin-based stress fibers, and Magatti et al. examined the complex polymer networks that occur in fibrin gels during blood clotting [66]. These works are related to ours in that they also involve initializing a random network of polymers, so that the algorithm described here to generate a random graph with specified statistical properties may be applicable. With regard to the image processing, our segmentation algorithm falls into the general class of thinning algorithms [67], which have been used elsewhere for biological applications such as extracting the structure of collagen gels [68, 69].

Although the present work has not been focused on the numerical details of the immersed boundary method, we nevertheless wish to mention a few challenges and potential research directions related to simulating red cells under flow. Whereas red cells in our bodies repeatedly experience many cycles of deformation over their lifespans, at present we are unable to simulate more than a few cycles because of the prohibitive computational cost of long simulations at high resolution. Continual deformations may also lead to numerical challenges in the form of long and skinny triangles in the mesh. In our simulations the aspect ratio, i.e. the ratio of the longest triangle edge length to the shortest triangle edge length, ranges from an average of 1.1 to an average of 1.3 in the tank treading simulations and of 3.3 in the optical tweezer simulations. The maximum aspect ratio over all triangles ranges from 1.8 initially to 13 in the tank treading simulations and to 920 in the optical tweezer simulations. Given the nature of the deformation in the optical tweezer simulation, it is physically reasonable that triangles become severely skewed in that case. This interplay of numerical and structural stability merits further study given that skewed triangles can decrease the accuracy of discretizations on the mesh [70].

Taken as a whole, this work describes a method to use tomographic data as a basis for simulating the effects of changes in cytoskeletal structure and dynamics on how red cells respond to different flow conditions. By applying this framework to a wider selection of tomographic samples, we believe it can provide a more detailed understanding of the cytoskeleton and its role in disorders affecting red cell fluid mechanics.

## Supporting information

S1 Text Appendix. (PDF)

S1 Video Watershed algorithm for extracting statistical properties from tomograms. (MP4)

S2 Video Simulation of red cell in shear flow, side view. (MP4)

S3 Video Simulation of red cell in shear flow, top view. (MP4)

S4 Video The distribution of edge lengths in the spectrin network changes over the course of breathing oscillations in shear flow. (MP4)

S5 Video Close-up of the dynamic spectrin network. (MP4)

S6 Video Optical tweezer simulation. (MP4)
