# Supplementary material for: Image-based model of the spectrin cytoskeleton for red blood cell simulation
Source: PLoS Comput Biol. 2017 Oct 9;13(10):e1005790. doi: 10.1371/journal.pcbi.1005790 (PMC5654263; doi:10.1371/journal.pcbi.1005790)
Supplement: S1 Text — (PDF) [file pcbi.1005790.s001.pdf]

Appendix

Entropic spring model

Spectrin tetramers consist of $N = 38$ linked monomers and have been measured to have a persistence length l_p of 10–20 nm and a mean contour length L of 200 nm [1]. We use these values in our simulations, taking $l_p = 20$ nm.

Let $\mathbf{r}$ denote the end-to-end vector of a polymer. A commonly-used linear approximation to the freely jointed chain model [1–4] yields the following probability density function $\sigma(D)$ for the end-to-end distance $D = \|\mathbf{r}\|$:

$$\sigma(D) = 4\pi(3/(4\pi L l_p))^{2/3} D^2 \exp\left(-3D^2/(4Ll_p)\right) \tag{1}$$

with the corresponding free energy given by

$$A(\mathbf{r}) = -k_B T \left(\frac{-3\|\mathbf{r}\|^2}{4Ll_p}\right) + C, \tag{2}$$

where C is a constant independent of $\mathbf{r}$. The force is given by $\mathbf{F} = -\nabla A$, which gives

$$\mathbf{F}_{\text{linear}}(\mathbf{r}) = -\frac{3k_B T}{2Ll_p}\mathbf{r}, \tag{3}$$

i.e. a Hookean spring with zero rest length and spring constant $k = 3k_B T/(2Ll_p)$. Since the source of the elasticity is purely combinatorial, i.e. there are many more polymer conformations in which the end-to-end length is small rather than large, this is known as an entropic spring model. Although (3) displays reasonable behavior at small extensions [1], it breaks down at extensions approaching the contour length. Whereas the end-to-end distance of a polymer consisting of finitely-many monomers is at most Nl_k, the linear approximation above allows in principle for infinite extensibility. The phenomenological worm-like chain model corrects this by introducing a divergent force at finite extension [5]. To represent each spectrin polymer we therefore use the worm-like chain model, which has the force-extension relationship

$$\mathbf{F}_{\text{wlc}}(\mathbf{r}) = F_{\text{wlc}}(\|\mathbf{r}\|)\frac{\mathbf{r}}{\|\mathbf{r}\|}, \qquad F_{\text{wlc}}(\|\mathbf{r}\|) = \frac{k_B T}{l_p}\left(\frac{1}{4}\left(1 - \frac{\|\mathbf{r}\|}{L}\right)^{-2} - \frac{1}{4} + \frac{\|\mathbf{r}\|}{L}\right). \tag{4}$$

The one case in which we use $\mathbf{F}_{\text{linear}}$ is for the test of red cells with dynamic networks subject to prescribed strain in the section titled “Simulating response to flow and applied strain”. When using the worm-like chain model, the number of irreversibly broken edges is difficult to detect above the noise of network dynamics, since the divergent force prevents edges from approaching the contour length (see the section below titled “Dynamic connections”). Note that $\mathbf{F}_{\text{wlc}}$ agrees with $\mathbf{F}_{\text{linear}}$ in the limit of small extensions, and arbitrarily large extensions do not occur when there is irreversible breakage beyond a threshold.

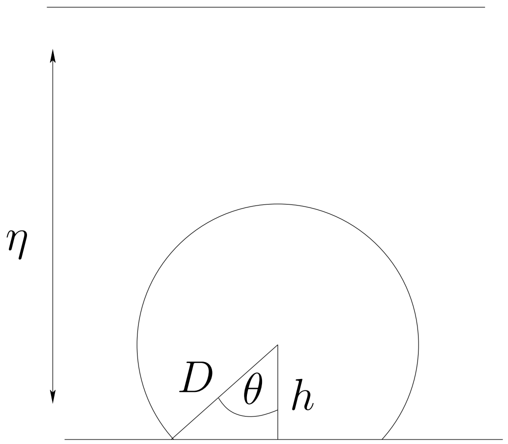

Fig A. Cross-section showing the intersection between a slab of thickness η and a sphere of radius $D < \eta/2$.

Density through slab

Given a slab with thickness η, we wish to compute the vertically-averaged node distance PDF $\varrho(D)$, defined in the section "Generating the cytoskeleton" of the Main Text by $\int_a^b \varrho(D)\mathrm{d}D = \Pr(D_{ij} \in (a,b))$, where D_{ij} is the distance between any two nodes i and j. As before, the nodes are assumed to be uniformly distributed through the slab. Throughout this appendix, we ignore the effects of edges in the horizontal directions. As $\eta \to \infty$ we should have $\varrho(D) \to 4\pi D^2/V$, where V is the volume of the slab. However, we are interested in the case of a thin slab with $\eta \ll \infty$, so the effects of finite η must be accounted for. We will consider several cases. First, take $D < \eta/2$, so that there is at most one intersection between a sphere of radius D centered inside the slab and the slab boundary. If the sphere is centered at a height $h > D$ above the lower slab boundary, it is fully contained within the slab. If $h < D$, the sphere intersects the lower boundary at some angle θ (see Fig A). The excluded solid angle Ω is given by

$$\Omega = \int_0^\theta \int_0^{2\pi} \sin\theta' \mathrm{d}\phi\, \mathrm{d}\theta' = -2\pi \cos\theta' \Big|_0^\theta = 2\pi(1-\cos\theta), \tag{5}$$

which implies that the excluded surface area is $2\pi D^2(1-\cos\theta)$. The vertically-averaged density is therefore given by

$$\varrho(D) = \frac{1}{V}\frac{1}{\eta/2}\left(\int_0^D (4\pi D^2 - 2\pi D^2(1-\cos\theta))\mathrm{d}h + \int_D^{\eta/2} 4\pi D^2 \mathrm{d}h\right), \tag{6}$$

where we have used the symmetry of the top and bottom halves of the slab. Since $\cos\theta = h/D$,

$$\begin{aligned} \varrho(D) &= \frac{1}{V}\left(4\pi D^2 - \frac{1}{\eta/2}\left(\int_0^D 2\pi D^2(1-h/D)\mathrm{d}h\right)\right) \\ &= \frac{4\pi D^2}{V}\left(1 - \frac{D}{2\eta}\right). \end{aligned} \tag{7}$$

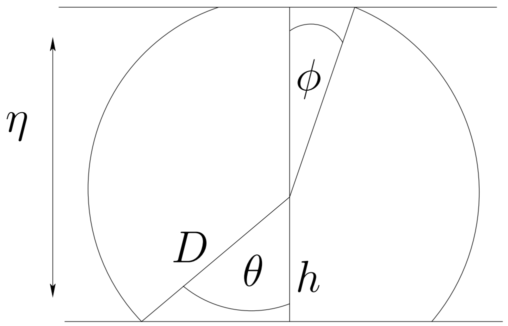

Fig B. Cross-section showing the intersection between both boundaries and the sphere of radius D such that $\eta/2 < D < \eta$.

Next, take $\eta/2 <= D < \eta$ so that the sphere may intersect with at least one and possibly both slab boundaries. Assume without loss of generality that the sphere intersects the lower boundary. Let θ be as above and, in the case that the sphere intersects with the upper boundary, define ϕ to be the associated angle of intersection (see Fig B). Now, we must account for potentially two excluded regions of the sphere, which yields

$$\begin{aligned}\varrho(D) &= \frac{1}{V}\frac{1}{\eta/2}\left(\int_0^{\eta-D}(4\pi D^2 - 2\pi D^2(1-\cos\theta))\mathrm{d}h\right.\\ &\quad\left.+\int_{\eta-D}^{\eta/2}(4\pi D^2 - 2\pi D^2(1-\cos\theta) - 2\pi D^2(1-\cos\phi)\mathrm{d}h\right)\\ &= \frac{4\pi D^2}{V}\left(1-\frac{1}{\eta}\int_0^{\eta/2}(1-h/D)\mathrm{d}h - \frac{1}{\eta}\int_{\eta-D}^{\eta/2}(1-(\eta-h)/D)\mathrm{d}h\right)\\ &= \frac{4\pi D^2}{V}\left(1-\left(\frac{1}{2}-\frac{\eta}{8D}\right)-\left(-\frac{1}{2}+\frac{\eta}{8D}+\frac{D}{2\eta}\right)\right)\\ &= \frac{4\pi D^2}{V}\left(1-\frac{D}{2\eta}\right).\end{aligned} \tag{8}$$

Note that the expression above is identical to the formula obtained for $D < \eta/2$. Finally, suppose that $D > \eta$. In this case, there is always an intersection with both upper and lower boundaries for all h and

$$\begin{aligned}\varrho(D) &= \frac{1}{V}\frac{1}{\eta/2}\left(\int_0^{\eta/2}(4\pi D^2 - 2\pi D^2(1-\cos\theta) - 2\pi D^2(1-\cos\phi))\mathrm{d}h\right)\\ &= \frac{4\pi D^2}{V}\frac{1}{\eta}\int_0^{\eta/2}\left(\frac{h}{D}+\frac{\eta-h}{D}\right)\mathrm{d}h\\ &= \frac{4\pi D^2}{V}\frac{\eta}{2D}\\ &= \frac{2\pi D\eta}{V}.\end{aligned} \tag{9}$$

In summary, $\varrho(D)$ is a piecewise function satisfying

$$\varrho(D) = \begin{cases} 4\pi D^2/V\left(1-\frac{D}{2\eta}\right)) & \text{if } D \le \eta,\\ 2\pi D\eta/V & \text{if } D > \eta.\end{cases} \tag{10}$$

Dynamic connections

Here, we extend our model to allow for dynamic connections that change over time. The dynamics of network connections are governed by rate constants $k_{\text{on}}(D)$ and $k_{\text{off}}(D)$ of formation and breakage, respectively. We assume that the rate constant of breaking is independent of distance so that $k_{\text{off}}(D) \equiv k_{\text{off}}$. We next show how to choose the function $k_{\text{on}}(D)$ that results in a steady-state distribution of edge lengths. Recall from the section "Generating the cytoskeleton" of the Main Text that $p(D)$ is the probability that a pair of nodes separated by a distance D is connected by an edge. Now letting $p = p(D,t)$ also depend on time, the dynamics of p for a cell at rest follow

$$\frac{\partial p}{\partial t} = k_{\text{on}}(1-p) - k_{\text{off}}\, p. \tag{11}$$

If we specify a constant rate of breaking $k_{\text{off}}(D) \equiv k_{\text{off}}$, we may use the steady-state condition

$$k_{\text{on}}(D) = k_{\text{off}} \frac{p(D)}{1-p(D)}, \tag{12}$$

and the form of $p(D)$, derived in the section "Generating the cytoskeleton" of the Main Text, to set $k_{\text{on}}(D)$. Note that (12) is only used to derive the rate constant $k_{\text{on}}(D)$ of edge formation and we assume $p(D)$ does not depend on time in all our simulations. The assumption that k_{off} is independent of D is arbitrary–the remodeling of spectrin is still not well-understood and there is insufficient experimental data to discriminate between competing models of network dynamics. However, for the purpose of this work the rate of remodeling matters more than its specific form and we believe the same qualitative effects would result for other models of network dynamics as well.

In our model the network dynamics are handled in a stochastic manner. To check whether new bonds are formed, for each eligible pair of points (i.e. not already connected and with separation $D < D_{\text{max}}$) we generate a random number uniformly between 0 and 1 at each time step. We draw a new edge between points if the corresponding random number is less that $k_{\text{on}}(D)\Delta t$. To determine whether two nodes become disconnected, we generate random numbers for each edge and remove that edge if the random number is less that $k_{\text{off}}\Delta t$. For computational efficiency, we recycle the binning of nodes into boxes used for generating the model cytoskeleton in the section "Generating the model cytoskeleton on a triangulated surface" of the Main Text. That is, at each time step, we update the boxes containing the nodes using methods described in [6]. This leads to a considerable computational savings, since when making new connections only pairs of nodes within the same and adjacent boxes must be checked.

To characterize the viscoelastic behavior of this dynamic network we simulate two tests of viscoelasticity on a remodeling network of worm-like chains. This is done on a 2D patch of cytoskeleton. The network is expected to behave like a dashpot representing the viscosity of the ambient fluid in parallel with a Maxwell element, i.e. a spring in series with another dashpot. The spring and dashpot components of the Maxwell element represent the elastic polymers and the viscous effect of remodeling, respectively. This is referred to as a Fluid 1 element in [7], in which the authors systematically study the response of 3D cytoskeletal networks made up of different viscoelastic elements.

Following the descriptions of viscometer tests in [7, 8], we first simulate a stress relaxation test in a viscometer by shearing the network and observing how the force required to maintain the shear decays over time (Fig C). To do this, a 2D random network made up of $N = 1,200$ nodes along with its corresponding triangulation are generated as in the section "Elastic response of random graphs" of the Main Text and then subjected to a prescribed shear strain. Nodes located in layers on the top and bottom boundaries are fixed in place, while the interior nodes move according to overdamped dynamics determined by worm-like chain and area-preserving forces. To

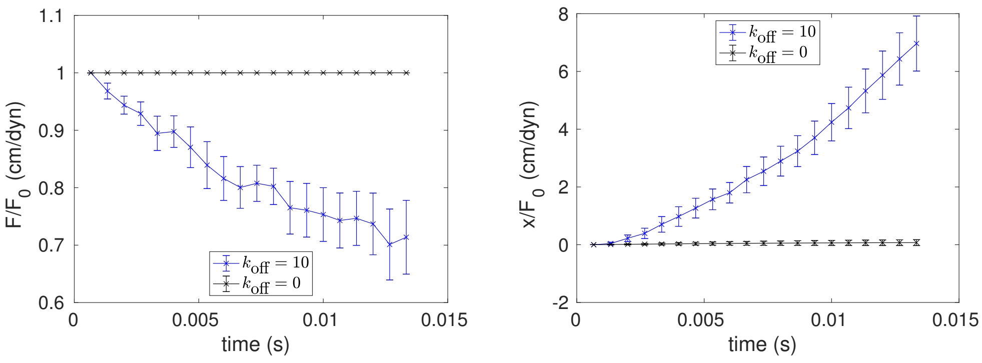

(a) (b)

Fig C. Relaxation and creep tests. (a) The force held by the random spring network decays with a timescale of ≈ 0.04 s when the network remodels with $k_{\text{off}} = 10$ s^{-1}. (b) In the presence of remodeling the random spring network undergoes plastic deformation, or creep.

separate the timescale of the fluid from the timescale of remodeling, the network is fixed with a static connectivity and relaxed to equilibrium before any bonds are allowed to break and reform. From the ratio of the equilibrium force and displacement, the material has an effective elastic modulus of $k \approx 0.02$ dyn/cm. Once network dynamics are turned on, the force decays with characteristic timescale of ≈ 0.04 s (Fig C(a)). Since this timescale is given by η_1/k for a Fluid 1 element, where η_1 is the dashpot viscosity in the Maxwell element [7], i.e. the effective viscosity of network we find $\eta_1 \approx 8 \cdot 10^{-4}$ g/s for the parameters used.

Next, we simulate a creep test in which, rather than a prescribed shear strain, we subject the network to a constant external force. The network is prepared as above. Nodes in a thin strip on the bottom boundary are held in place, but instead of prescribing the positions of the nodes on the top boundary, they are subjected to a constant force. In the case of a purely elastic material, the top nodes would not move past their equilibrium positions. This is indeed observed when the network is not allowed to remodel (Fig C(b)). However, with remodeling, there is a plastic deformation, or *creep*, and the nodes drift gradually in the direction of the applied force. The rate of drift is equal to $(\eta_1 + \eta_2)^{-1}$, the inverse of the sum of dashpot viscosities in the Fluid 1 element [7]. After the initial transient, we find $\eta_1 + \eta_2 = 1.2 - 2.0 \cdot 10^{-3}$ g/s.

Putting these calculations together gives the estimate $\eta_2 = 0.4 - 1.2 \cdot 10^{-3}$ g/s for the viscosity on the whole structure due to the fluid. This result is self-consistent, since the net friction of $1,200 \cdot 3.3 \cdot 10^{-7} = 4.0 \cdot 10^{-4}$ g/s, calculated by multiplying the friction coefficient $\zeta = 3.3 \cdot 10^{-7}$ times the total number of nodes, lies within this range.

To justify using a linear polymer force model instead of a worm-like chain for the optical tweezer simulations in the section "Simulating response to flow and applied strain" of the Main Text, here we compare differences in irreversible breakage events on a 2D patch of cytoskeleton using both the worm-like chain and linear spring models. The two ends of the domain are pulled apart at a fixed rate, which stretches out the material in a manner reminiscent of optical tweezers experiments. We consider a patch of cytoskeleton on a 2D inextensible sheet being stretched out in the presence of network dynamics for both the linear spring and worm-like chain models. Here we use a threshold of $0.9L$ for irreversible breakage. Upon simulation, we find many more broken edges when using the linear spring model, which is expected given the stiffening of the worm-like chain model at large extensions. Although the edge-length distribution extracted by the image processing has a small tail beyond the irreversible breakage

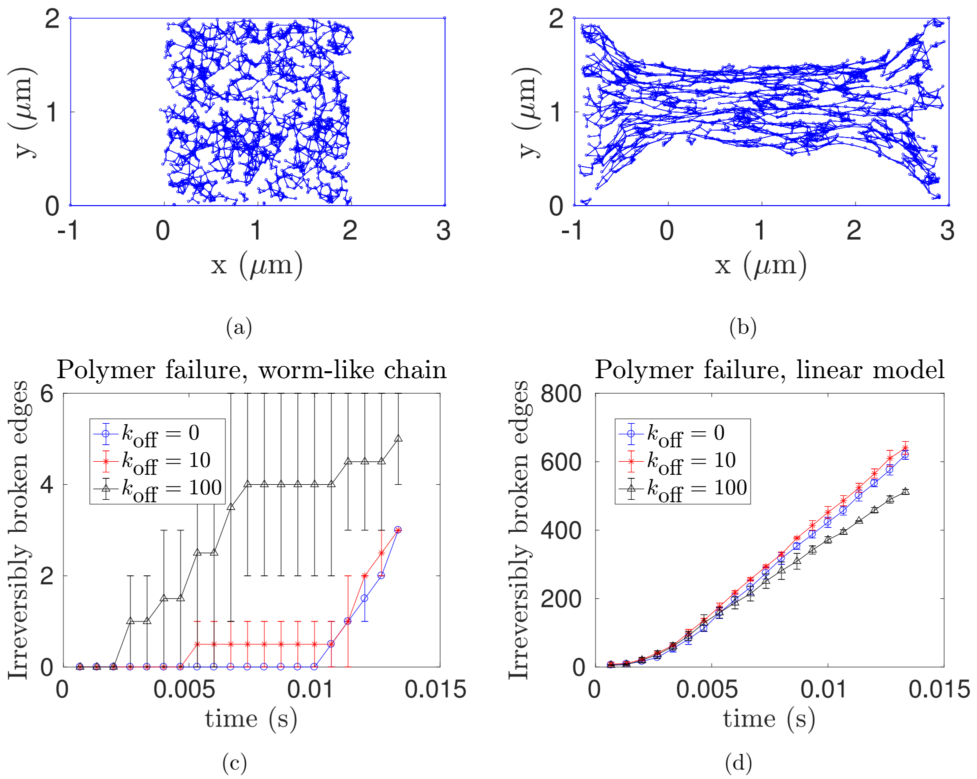

Fig D. Network dynamics on a 2D patch of cytoskeleton. (a) Initial condition, (b) Final state after stretch, (c) Worm-like chain polymer model, (d) Linear polymer model.

threshold, there are values of $D > 0.9L$ for which $\sigma(D) \neq 0$. This gives a small chance of an edge being formed under network dynamics that instantly breaks. Thus network dynamics leads trivially to a background increase in the number of broken edges, but we are interested in the signal of irreversible breakage events above this background. As shown in the Fig D, we can detect this signal using the linear elasticity model, whereas we are currently unable to do so using the worm-like chain model because of the small number of events and large error bars.

Immersed boundary method

The immersed boundary formulation is a mathematical description of fluid-structure interaction that combines the Navier-Stokes equations with the dynamics of an immersed elastic material (see for instance [9, 10] and references therein). Fluid with density ρ and viscosity ν is assumed to fill a domain, and the fluid is described in cartesian coordinates $\mathbf{x}$ by a velocity field $\mathbf{u}(\mathbf{x}, t)$ and pressure field $p(\mathbf{x})$. The domain also contains an elastic material, which is convenient to describe in Lagrangian coordinates $\mathbf{q}$ that denote material points. There is a function $\mathbf{X}(\mathbf{q}, t)$ that returns the Cartesian position of material point $\mathbf{q}$ at time t, and the elastic energy of the material can be computed as a functional of the deformed configuration so that $E = E[\mathbf{X}(\cdot, t)]$. This gives rise to a force density $\mathbf{F}(\mathbf{q}, t) = -\delta E/\delta \mathbf{X}$, where $\delta/\delta \mathbf{X}$ denotes the variational derivative with respect to the configuration of the elastic material.

The equations that characterize the immersed boundary formulation are as follows:

$$\rho\left(\frac{\partial \mathbf{u}}{\partial t}+\mathbf{u}\cdot\nabla\mathbf{u}\right)+\nabla p=\nu\Delta\mathbf{u}+\mathbf{f}, \tag{13}$$

$$\nabla\cdot\mathbf{u}=0, \tag{14}$$

$$\mathbf{f}(\mathbf{x},t)=\int\mathbf{F}(\mathbf{q},t)\delta\left(\mathbf{x}-\mathbf{X}(\mathbf{q},t)\right)\mathrm{d}\mathbf{q}, \tag{15}$$

$$\frac{\partial \mathbf{X}}{\partial t}=\mathbf{U}(\mathbf{q},t)=\int\mathbf{u}(\mathbf{x},t)\delta\left(\mathbf{x}-\mathbf{X}(\mathbf{q},t)\right)\mathrm{d}\mathbf{x}, \tag{16}$$

$$\mathbf{F}(\mathbf{q},t)=-\delta E[\mathbf{X}(\cdot,t)]/\delta\mathbf{X}. \tag{17}$$

Equations (13)-(14) are the Navier-Stokes equations and (17) gives the force density on the membrane in Lagrangian coordinates. Equations (15)-(16) are the interactions equations that couple the fluid and elastic material. The Lagrangian force density is convolved against the Dirac delta function $\delta(\mathbf{x})$, giving rise to the force density $\mathbf{f}(\mathbf{x},t)$ in cartesian coordinates that appears in (13). In our case, the Lagrangian force involves one contribution from the membrane and another from the cytoskeleton. At a discrete point on the cytoskeleton, the force is computed by

$$\mathbf{F}_k=\sum_j\mathbf{F}_{\mathrm{wlc}}(\mathbf{X}_j-\mathbf{X}_k), \tag{18}$$

where the sum is over all points $\mathbf{X}_j$ attached by an edge in the network to $\mathbf{X}_k$. At a discrete point on the membrane, on the other hand, the force is defined in terms of the energy W:

$$\mathbf{F}_k=-\nabla_{\mathbf{X}_k}W, \tag{19}$$

where $W=W_{\mathrm{bulk}}+W_{\mathrm{bend}}$ and $\nabla_{\mathbf{X}_k}$ denotes the gradient of the energy with respect to the position $\mathbf{X}_k$. We remark that $\mathbf{F}_k$ is the force, not the force density, applied at point $\mathbf{X}_k$ to the fluid. Finally, to make the formulation complete, we specify the membrane energy via

$$W_{\mathrm{bulk}}=\frac{\kappa}{2}\int_{\mathbf{Z}}(J-1)^2\,\mathrm{d}a \tag{20}$$

$$W_{\mathrm{bend}}=\frac{k_{\mathrm{bend}}}{2}\int_S H^2\mathrm{d}a, \tag{21}$$

where $\mathbf{Z}$ is the reference configuration, H is the total curvature, i.e. the sum of the two principal curvatures, and $k_{\mathrm{bend}}=2.0\times10^{-19}$ J [11] is the bending rigidity. The bulk modulus κ and Jacobian J are as in the section titled "Elastic response of random graphs". The total force is obtained by summing over all points on the membrane and cytoskeleton. See [10,12] for details regarding how these forces are computed in practice.

The elastic material moves at the local fluid velocity according to (16). The immersed boundary equations describe the nature of the coupling between the membrane and cytoskeleton, which are treated as independent elastic structures in our simulations that are both immersed in the same ambient fluid. The forces they exert are both spread onto cartesian coordinates so that $\mathbf{f}(\mathbf{x},t)$ is the resultant force, which determines the fluid velocity in which both structures move. Because the membrane and cytoskeleton initially lie on the same surface (see Fig E), they stay together since they are advected in the same velocity field. Diffusion of cytoskeletal proteins and slip relative to the membrane can be included within the immersed boundary framework [13,14], and adding these effects would make the model even more realistic and allow for simulating an even larger range of observed phenomena, such as the nontrivial interplay between diffusion of cytoskeletal proteins and disruptions in both

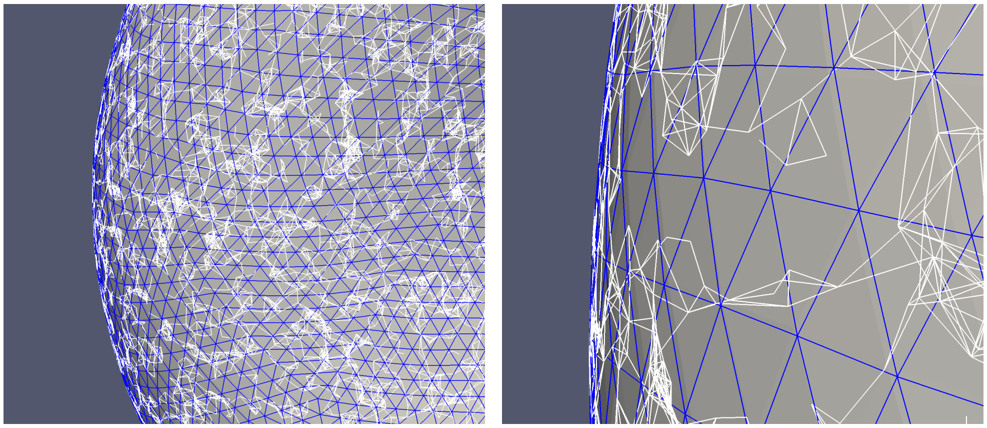

(a) (b)

Fig E. Overlay of the triangulated membrane and discrete cytoskeleton. (a) The triangulation representing the membrane (triangle edges in blue) and the superimposed cytoskeleton (spectrin edges in white), (b) A close-up of the same structure.

vertical connections to the membrane and horizontal cytoskeletal connections observed in computational [15] and experimental [16] studies.

In the present context of the continuous problem, writing the interpolation equation as above with convolution against a Dirac delta function is superfluous; the velocity field $\mathbf{u}$ needs only to be evaluated at a material point $\mathbf{X}(\mathbf{q}, t)$ to find the velocity there. However, this equation becomes useful once the equations are discretized, since replacing the Dirac delta function by a regularization yields a numerical method for performing the interpolation. The choice of regularized delta function is the key ingredient of the immersed boundary method; it should interpolate smooth functions accurately and have finite support for computational efficiency. In the simulations presented here we have used a delta function with a support of 4 gridpoints in each spatial dimension. For further details, see [9, 10, 17].

In our simulations, we have taken water to be the ambient fluid, so that $\rho = 1\,\mathrm{g/cm^3}$ and $\nu = 1.2$ cP, and the domain to be a rectangular box of size 10 µm ×10 µm ×20 µm with periodic boundary conditions in each direction. A discretization of the box with either $128 \times 128 \times 256$ or $256 \times 256 \times 512$ is used, which results in an equal grid spacing Δx in each direction. We have found it necessary to take a timestep Δt with $\Delta t = (0.01 - 0.1)\Delta x$ to ensure stability. A predictor-corrector timestepping scheme is used [10], and the linear system resulting from discretizing the Navier-Stokes equations are solved with fast Fourier transforms. The surface of the red cell is represented by a triangulated surface composed of approximately 20,000 triangles. The method has been implemented with shared memory parallelism on the GPU using Python and OpenCL. Typical simulations of cells with static networks take 1–7 days of continuous computing time depending on the resolution of the fluid grid, whereas typical simulations of cells with dynamic networks take several days to weeks.

References

1. Boal D. Mechanics of the Cell. Cambridge University Press; 2012.

2. Schwartz R. Biological modeling and simulation: a survey of practical models, algorithms, and numerical methods. MIT Press; 2008.

3. Barrat JL, Hansen JP. Basic concepts for simple and complex liquids. Cambridge University Press; 2003.

4. Doi M, Edwards S. The theory of polymer dynamics. Clarendon: Oxford. 1986;.

5. Marko JF, Siggia ED. Stretching DNA. Macromolecules. 1995;28(26):8759–8770.

6. McQueen DM, Peskin CS. Shared-Memory Parallel Vector Implementation of the Immersed Boundary Method for the Computation of Blood Flow in the Beating Mammalian Heart. The Journal of Supercomputing. 1997;11:213–236. doi:10.1023/A:1007951707260.

7. Wróbel JK, Cortez R, Fauci L. Modeling viscoelastic networks in Stokes flow. Physics of Fluids. 2014;26(11):113102.

8. Fung Y. Biomechanics: mechanical properties of living tissues. Springer Science & Business Media; 2013.

9. Peskin CS. The immersed boundary method. Acta Numerica. 2002;11.

10. Fai TG, Griffith BE, Mori Y, Peskin CS. Immersed Boundary Method for Variable Viscosity and Variable Density Problems Using Fast Constant-Coefficient Linear Solvers I: Numerical Method and Results. SIAM Journal on Scientific Computing. 2013;35(5):B1132–B1161.

11. Mohandas N, Evans E. Mechanical Properties of the Red Cell Membrane in Relation to Molecular Structure and Genetic Defects. Annual Review of Biophysics and Biomolecular Structure. 1994;23:787–818.

12. Wu CH, Fai TG, Atzberger PJ, Peskin CS. Simulation of Osmotic Swelling by the Stochastic Immersed Boundary Method. SIAM Journal on Scientific Computing. 2015;37(4):B660–B688.

13. Atzberger PJ, Kramer PR, Peskin CS. A stochastic immersed boundary method for fluid-structure dynamics at microscopic length scales. Journal of Computational Physics. 2007;224:1255–1292.

14. Kim Y, Peskin CS. 2–D Parachute Simulation by the Immersed Boundary Method. SIAM J Sci Comput. 2006;28(6):2294–2312.

15. Li H, Zhang Y, Ha V, Lykotrafitis G. Modeling of band-3 protein diffusion in the normal and defective red blood cell membrane. Soft Matter. 2016;12(15):3643–3653.

16. Kodippili GC, Spector J, Sullivan C, Kuypers FA, Labotka R, Gallagher PG, et al. Imaging of the diffusion of single band 3 molecules on normal and mutant erythrocytes. Blood. 2009;113(24):6237–6245.

17. Liu Y, Mori Y. Properties of discrete delta functions and local convergence of the immersed boundary method. SIAM Journal of Numerical Analysis. 2012;50:2986–3015.
